# Slow recovery from a disease epidemic in the spotted hyena, a keystone social carnivore

**DOI:** 10.1038/s42003-018-0197-1

**Published:** 2018-11-20

**Authors:** Sarah Benhaiem, Lucile Marescot, Marion L. East, Stephanie Kramer-Schadt, Olivier Gimenez, Jean-Dominique Lebreton, Heribert Hofer

**Affiliations:** 1 Department of Ecological Dynamics, Leibniz Institute for Zoo and Wildlife Research, Alfred-Kowalke-Strasse 17, D-10315 Berlin, Germany; 2 CEFE, CNRS, University Montpellier, University Paul Valéry Montpellier 3, EPHE, IRD, Montpellier, 34090 France; 3 Department of Ecology, Technische Universität Berlin, Rothenburgstr. 12, 12165 Berlin, Germany; 4 Department of Veterinary Medicine, Freie Universität Berlin, Oertzenweg 19b, Berlin, 14163 Germany; 5 Department of Biology, Chemistry, Pharmacy, Freie Universität Berlin, Takustr. 3, Berlin, 14195 Germany

## Abstract

Predicting the impact of disease epidemics on wildlife populations is one of the twenty-first century’s main conservation challenges. The long-term demographic responses of wildlife populations to epidemics and the life history and social traits modulating these responses are generally unknown, particularly for *K*-selected social species. Here we develop a stage-structured matrix population model to provide a long-term projection of demographic responses by a keystone social predator, the spotted hyena, to a virulent epidemic of canine distemper virus (CDV) in the Serengeti ecosystem in 1993/1994 and predict the recovery time for the population following the epidemic. Using two decades of longitudinal data from 625 known hyenas, we demonstrate that although the reduction in population size was moderate, i.e., the population showed high ecological ‘resistance’ to the novel CDV genotype present, recovery was slow. Interestingly, high-ranking females accelerated the population’s recovery, thereby lessening the impact of the epidemic on the population.

## Abstract (web summary)

Sarah Benhaiem et al. present a model of demographic responses by the spotted hyena to an epidemic of canine distemper virus in 1993–1994.They find that recovery from the outbreak was slow despite initial moderate mortality, and that high-ranking females sped up recovery by increasing the ecological resistance of the population.

## Introduction

Epidemics responsible for a decline in keystone species can alter ecosystem dynamics and diminish biodiversity by increasing the chance of extirpation of host populations, and possibly the extinction of species^1–4^. Human activities rapidly expand the geographical range and host species spectrum of pathogens, and epidemics caused by exotic pathogens in unexpected hosts are increasing^5^. Viruses are of particular concern as they evolve rapidly, yielding new strains and adaptations to novel hosts^3,5^.

We know little about the long-term demographic responses to infectious viral disease epidemics in wildlife species, particularly those with high maternal investment in a low number of offspring during a long lifespan (*K*-selected species), probably because longitudinal studies on such species are rare^6^. To characterise these demographic responses, we apply two terms from the field of ecology—ecological resistance: the impact of exogenous disturbance on the state of a system; and recovery: the endogenous process that pulls the disturbed system back towards equilibrium^7^. In social host species, the relationships between social traits and disease outcomes can be complex^8–10^. It is therefore unclear how social traits modulate the magnitude of the potential reduction in host population size during an epidemic, or the recovery time to pre-epidemic population size^11^.

There is a strong need for a robust predictive framework to assess the potential risk that epidemics pose to wildlife populations, to provide projections of the recovery of populations from epidemics and to identify factors modulating population responses, particularly for *K*-selected and social species. Here we show that stage-structured matrix population models^12,13^ are highly suitable for these purposes because they allow us to assess how demographic performance (in terms of fecundity, survival and reproductive value) is influenced by infection status, and how disease persistence and dynamics are influenced by host demography and social structure^14,15^. Such models can make full use of field data as input, i.e., state-specific parameters estimated by capture-mark-recapture (CMR) approaches^16^, and permit the calculation of the basic reproduction number (*R*_0_) of a pathogen^14,15^. *R*_0_ is a measure of the number of individuals infected by introducing a single infected individual into a susceptible population (over the course of its infectious period)^17^, i.e., a measure of maximum transmissibility, and describes whether an infectious disease will invade or fade out in a population.

Here, we applied a stage-structured matrix population model to a longitudinal dataset of 625 individually known female spotted hyenas *Crocuta crocuta* (hereafter hyenas) in the Serengeti National Park, Tanzania, spanning two decades. We investigated the population consequences of a canine distemper virus (CDV) epidemic^10,18^, caused by a novel genotype better adapted to non-canids such as hyenas and lions *Panthera leo* than to canids^19^. By quantifying and conducting a sensitivity analysis for *R*_0_ of this CDV strain for the first time in hyenas, we formally demonstrate that it was highly contagious among hyenas during the epidemic and that the most influential parameter to decrease *R*_0_ was the probability of becoming a socially high-ranking breeder. CDV infection during the epidemic decreased the survival of cubs and subadults, particularly low-ranking ones. The projected hyena population showed a slow recovery, taking at least 16 years to return to pre-epidemic levels. Population growth rate and *R*_0_ were particularly sensitive to changes in the demographic contribution of high-ranking females, in terms of their probabilities of recruitment, survival and maintenance of high rank. Thus, high-ranking females increased the ecological resistance of the hyena population and helped to accelerate its recovery, thereby favouring the fadeout of the CDV epidemic. To our knowledge, this is the first study in a wildlife host to demonstrate that the interaction of host demographic performance, social status and infection state drive both *R*_0_ and the population response to a major epidemic.

## Results

### Demographic, social and infection parameter estimates

Each year, each female was assigned a demographic (cub, subadult, adult breeder or adult non-breeder), a social (high or low social status) and an infection (susceptible, infected or recovered) state. We fitted a multi-event CMR (MECMR) model that accounted for uncertainty on the assignment of infection states, i.e., we included animals with unknown infection states^10^. We focused on three periods: pre-epidemic (pre-epidem*;* 1990–1992), epidemic (epidem; 1993–1994), and post-epidemic (post-epidem*;* 1995–1999). The virulent CDV strain was not detected in any of our study clans after 1997^10^ and it was not detected in any host in the Serengeti ecosystem after 1999^19^.

The annual apparent survival probability (*ϕ*) varied over time, being highest during pre-epidem and lowest during epidem for all states, as the effect of periods was additive to the effect of given states (Table 1). The transition probability from the susceptible to the infected state (*β*, the infection probability) also varied across periods; it was highest during epidem and lowest during pre-epidem. CDV infection requires contact with aerosol droplets and body fluids from virus shedding individuals^20^. High-ranking subadults, breeders and non-breeders had a higher infection probability than low-ranking ones (Table 1), a consequence of their higher contact rates^10,21^. As described previously^10,20^, CDV infection decreased survival only among cubs and subadults but not adults (whether breeders or non-breeders, Table 1). Cubs of low-ranking mothers had a higher infection probability and were less likely to survive CDV infection than those of high-ranking mothers. This is thought to be a consequence of their poorer body condition and lower allocation of resources to immune processes^10^. High-ranking females were more likely to become breeders (*ψ*, the probability of assuming a breeder state) than low-ranking females. High-ranking females were less likely than low-ranking ones to maintain their social status (*r*, the probability of maintaining the current social state). Estimates of all parameters for each period, which were used as input for the subsequent stage-structured matrix population model, are shown in Table 1. Probabilities of detection and assignment of infection states were assumed to be constant throughout the study period.

**Table 1:** Input parameter values for the matrix population model

Process	Parameter and notation	m.l.e ± s.e.m		
		Pre-epidem	Epidem	Post-epidem
Survival	H susceptible cubs—*ϕ*_CHS_	0.90 ± 0.04	0.84 ± 0.07	0.86 ± 0.06
	L susceptible cubs—*ϕ*_CLS_	0.86 ± 0.07	0.78 ± 0.10	0.81 ± 0.08
	H infected cubs—*ϕ*_CHI_	0.79 ± 0.06	0.67 ± 0.07	0.72 ± 0.06
	L infected cubs—*ϕ*_CLI_	0.62 ± 0.08	0.48 ± 0.08	0.54 ± 0.08
	H susceptible subadults—*ϕ*_SAHS_	0.97 ± 0.04	0.95 ± 0.06	0.96 ± 0.05
	L susceptible subadults—*ϕ*_SALS_	0.90 ± 0.12	0.83 ± 0.19	0.86 ± 0.16
	H and L infected and recovered subadults—*ϕ*_SAIR_	0.69 ± 0.04	0.56 ± 0.05	0.61 ± 0.03
	H and L non-breeders—*ϕ*_NB_	0.86 ± 0.02	0.78 ± 0.03	0.82 ± 0.02
	H and L breeders—*ϕ*_B_	0.95 ± 0.01	0.91 ± 0.02	0.93 ± 0.01
Infection	H cubs—*β*_CH_	0.23 ± 0.09	0.96 ± 0.02	0.67 ± 0.16
	L cubs—*β*_CL_	0.45 ± 0.16	0.99 ± 0.01	0.85 ± 0.10
	H subadults, non-breeders and breeders—*β*_H_	0.10 ± 0.04	0.90 ± 0.05	0.42 ± 0.16
	L subadults, non-breeders and breeders—*β*_L_	0.02 ± 0.01	0.66 ± 0.12	0.13 ± 0.08
Demography	H subadults becoming H breeders—*ψ*_SAH_		0.04 ± 0.02	
	L subadults becoming L breeders—*ψ*_SAL_		0.01 ± 0.01	
	H non-breeders becoming H breeders—*ψ*_NBH_		0.68 ± 0.02	
	L non-breeders becoming L breeders—*ψ*_NBL_		0.60 ± 0.03	
	H breeders remaining H breeders—*ψ*_BH_		0.49 ± 0.02	
	L breeders remaining L breeders—*ψ*_BL_		0.45 ± 0.03	
Social	H remaining H—*r*_H_		0.94 ± 0.01	
	L remaining L—*r*_L_		0.97 ± 0.00	
Other	Average sex ratio in the population—*sex.ratio*		0.52	
	Average litter size in the population—*litter.size*		1.53	

Demographic states were abbreviated as *C* cubs, *SA* subadults, *B* adult breeders, or *NB* adult non-breeders, social states as *H* high status or *L* low status and infection states as *S* susceptible, *I* infected or *R* recovered. Note that the parameter values for demography and social were calculated across all three periods (pre-epidem, epidem and post-epidem)

Maximum likelihood estimate and associated standard error (m.l.e ± s.e.m.) of the probabilities of surviving, becoming infected, becoming a breeder, and maintaining the current social state in female hyenas during each period, as estimated via the multi-event capture-mark-recapture (MECMR) model. Sex ratio and litter size were estimated differently

### Population and CDV dynamics

**Fig. 1:**
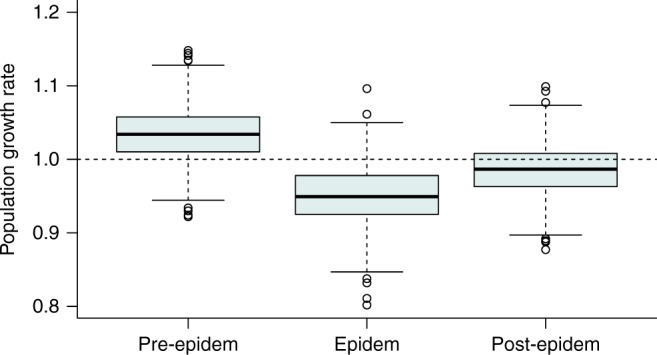
Population growth rate (*λ*) of female spotted hyenas. Data shown are for female spotted hyenas in the Serengeti National Park during the three epidemic periods (pre-epidem: 1990–1992; epidem: 1993–1994; post-epidem: 1995–1999). Empty circles represent outliers, the boxes encompass the first to the third quartiles, inside the box the thick horizontal line shows the median and the whiskers are located at 1.5 × IQR (interquartile range) below the first quartile and at 1.5 × IQR above the third quartile

The population growth rate (*λ*) differed substantially between periods (Fig. 1). *λ* was highest during pre-epidem and lowest during epidem. The hyena population increased during pre-epidem but decreased during epidem and post-epidem (Fig. 1).

We estimated CDV *R*_0_ in a closed hyena population, based on our three study clans, and hence did not consider the spread of the non-canid strain in other non-canid species, such as the lion, which were infected with this strain during the epidemic^19^. We modified a previously developed approach for discrete time models^14,15^. Incorporating population growth rate into the calculation produced a measure of change in the rate of spread of the disease accounting for change in population size, i.e., measuring change in the incidence rate of the disease. We then estimated this modified version of *R*_0_ during the epidemic period. *R*_0_ was relatively high, equal to 5.69 ± 0.67, illustrating how CDV infection spread rapidly among hyenas during epidem.

### Sensitivity analyses of *λ* and *R*_0_

**Fig. 2:**
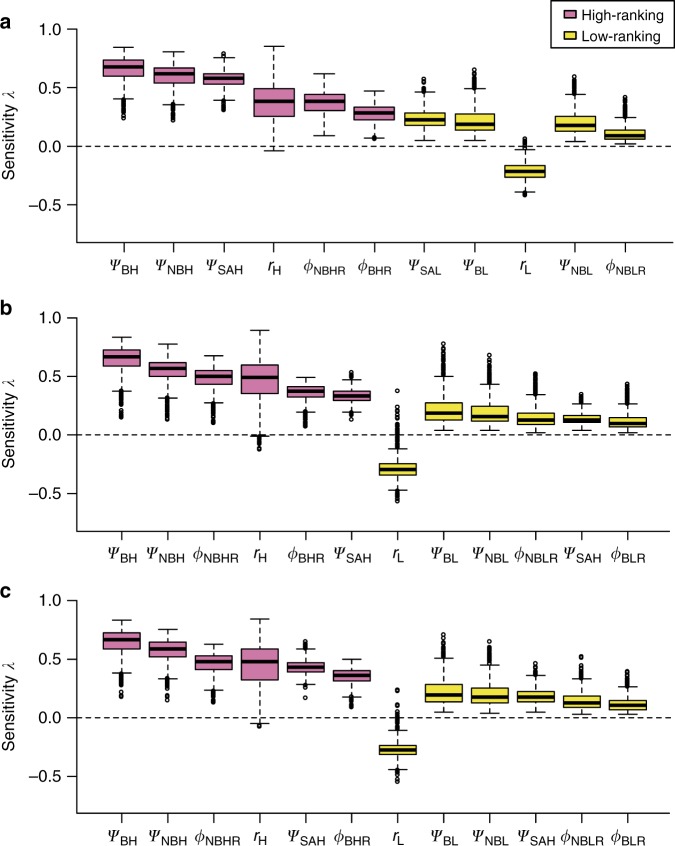
Sensitivity of the population growth rate (*λ*) to changes in vital rates in female hyenas during each period. **a** pre-epidemic, **b** epidemic and **c** post-epidemic. We only show the vital rates with effects on *λ* exceeding 10%, and order them according to their decreasing contribution to the absolute value of change of *λ*. Empty circles represent outliers, the boxes encompass the first to the third quartiles, inside the box the thick horizontal line shows the median and the whiskers are located at 1.5 × IQR (interquartile range) below the first quartile and at 1.5 × IQR above the third quartile. The pink boxes represent the vital rates for high-ranking females and the yellow boxes those for low-ranking ones. For the notation of parameters, see Table 1. Note that some parameters, such as the survival of high-ranking recovered non-breeder females *ϕ*_NBHR_,were not present as input parameter values in Table 1. This is because the function used to conduct the sensitivity analysis of *λ* considered each parameter of the symbolic matrix as unique in itself, even if input values were similar

To determine which vital rates (parameters) contributed most to changes in *λ* and *R*_0_, we conducted sensitivity analyses for *λ* during each period and for *R*_0_ during epidem. For *λ*, during all periods, the two most influential parameters were the probabilities that high-ranking breeders and non-breeders stayed or became high-ranking breeders (Fig. 2). In all periods, the probability of staying a high-ranking female had an important positive contribution on *λ* (Fig. 2). For all parameters and during each period, high-ranking females contributed substantially more to *λ* than low-ranking ones. The survival of non-breeders had a higher contribution than that of breeders because non-breeders were more likely to assume a breeder state than breeders at the next projection interval (Table 1). If breeders and non-breeders were allocated identical probabilities of assuming a breeder state, then the survival of breeders had a higher influence on *λ* than that of non-breeders.

**Fig. 3:**
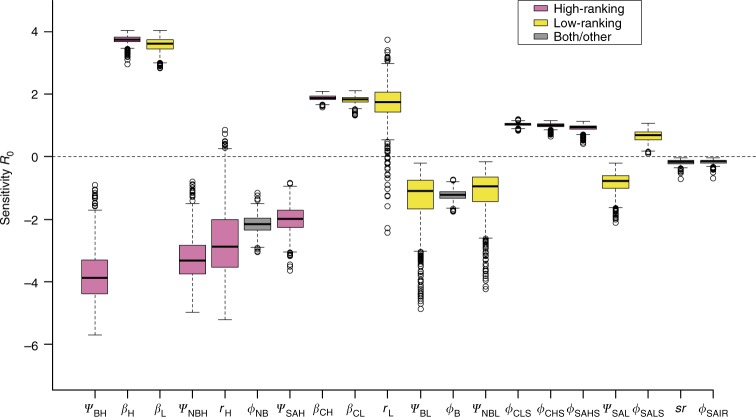
Sensitivity of *R*_0_ to changes in vital rates in female hyenas during the epidemic period. *R*_0_ is the basic reproduction number for the virulent 1993/1994 CDV strain in hyenas. We only show the vital rates with effects on *R*_0_ exceeding 10% and order them according to their decreasing contribution to the change in absolute value of *R*_0_. Empty circles represent outliers, the boxes encompass the first to the third quartiles, inside the box the thick horizontal line shows the median and the whiskers are located at 1.5 × IQR (interquartile range) below the first quartile and at 1.5 × IQR above the third quartile. As in Fig. 2, pink boxes represent the vital rates for high-ranking females and yellow boxes those for low-ranking ones. The grey boxes represent vital rates for both high and low-ranking combined or vital rates unrelated to rank. For the notation of parameters, see Table 1. Note that *sr* here represents sex ratio

The three most influential parameters to decrease *R*_0_ during epidem were increases in the probabilities that high-ranking breeders remained and high-ranking non-breeders became high-ranking breeders, and an increase in the probability of staying high-ranking across all demographic and infection states (Fig. 3). The most influential parameters to boost *R*_0_ were increases in the probabilities of becoming infected; the probability of staying low-ranking; and the survival of susceptible cubs and subadults (Fig. 3). As the infection probability was very high during epidem (Table 1), a high survival of susceptible cubs and subadults indicated a high infection risk. For each parameter except the survival of susceptible cubs, high-ranking females contributed substantially more than low-ranking ones to either an increase or decrease in *R*_0._

### Stable stage distribution (asymptotic values)

The proportions of female cubs, subadults, breeders and non-breeders varied little across periods (Supplementary Fig. 1). On average (±s.d.), breeders (0.35 ± 0.04) and non-breeders (0.34 ± 0.04) were more common than cubs (0.18 ± 0.03) or subadults (0.12 ± 0.02).

The proportions of low and high social states were similar and varied little across periods (Supplementary Fig. 2), with slightly more low-ranking females (0.54 ± 0.04) than high-ranking ones (0.46 ± 0.03). The higher proportion of low-ranking females was a consequence of a higher probability of transition from a high to a low social state than from a low to a high social state (see Table 1).

**Fig. 4:**
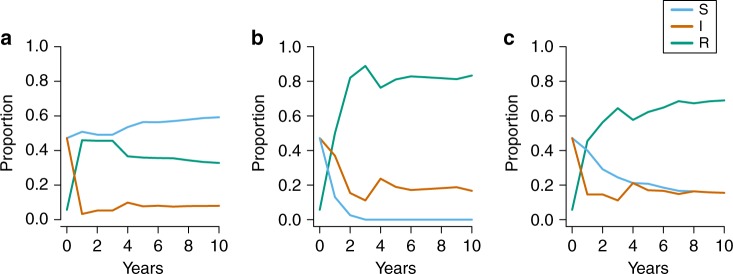
Dynamic projections of the proportion of different infection states and their convergence to a stable stage distribution. Projections are shown for medium term (first 10 years). We show dynamic projections for susceptible [S] (blue), infected [I] (orange) and recovered [R] (green) females (across all demographic and social states) during **a** pre-epidem, **b** epidem, **c** post-epidem. The starting values in each panel correspond to the (time invariant) initial state vector projection from the MECMR model. This figure illustrates which infection state predominates in each epidemic period, and how quickly each state converges to the stable stage distribution

The proportions of susceptible, infected and recovered females substantially varied across periods (Fig. 4 and Supplementary Fig. 3). Asymptotic, predicted values as expected from a stable stage distribution were as follows: during pre-epidem, the proportion of susceptible females was high (0.60 ± 0.02) and the proportions of infected and recovered were low (0.08 ± 0.01 and 0.31 ± 0.03, respectively). During epidem, the proportion of susceptible individuals was very low (0.01 ± 0.001), whereas the relative proportions of infected and recovered individuals was high (0.17 ± 0.002 and 0.82 ± 0.07, respectively). During post-epidem, there was a notable increase in the proportion of susceptible and a decrease in the proportion of recovered individuals (susceptible: 0.15 ± 0.01, infected: 0.16 ± 0.001, recovered: 0.70 ± 0.06).

### Transient dynamics

Figure 4 shows how the proportions of females in each infection state changed over the first 10 projected years and then reached a stable stage distribution. During epidem and post-epidem, the pool of susceptibles was rapidly depeleted, owing to the very high probabilities of infection (Table 1).

### Reproductive values

As is typical for the reproductive value, representing the expected current and future reproductive output^22^, it initially increased with age, that is, from cubs to subadults and also from subadults to breeders and non-breeders. Across study periods, the reproductive value of subadults was 2.2 times higher than that of cubs and the reproductive value of adults (pooled breeders and non-breeders) was 6.2 and 2.8 times higher than that of cubs and subadults, respectively (Supplementary Fig. 4). Among adults, the reproductive value of breeders was higher than that of non-breeders across periods (averaged values across periods: breeders: 17.4 ± 0.11, non-breeders 14.1 ± 0.16). Reproductive values varied little across periods (Supplementary Fig. 4).

High-ranking females, irrespective of their demographic state or infection state, had a reproductive value (31.6 ± 1.69) almost twice as large as that of low-ranking females (16.3 ± 1.22) across all periods, demographic states and infection states (Supplementary Fig. 5).

Across all periods, reproductive values of females were similar between different infection states (susceptible: 17.1 ± 1.46, infected: 16.5 ± 1.44, recovered: 14.3 ± 1.54, Supplementary Fig. 6). The lower value for recovered individuals, as compared to susceptible and infected ones, resulted from the fact that the recovered state did not include any cub (because cubs were never diagnosed as recovered in our original data set^10^), whereas both the susceptible and the infected states included cubs.

### Ecological resistance and recovery

**Fig. 5:**
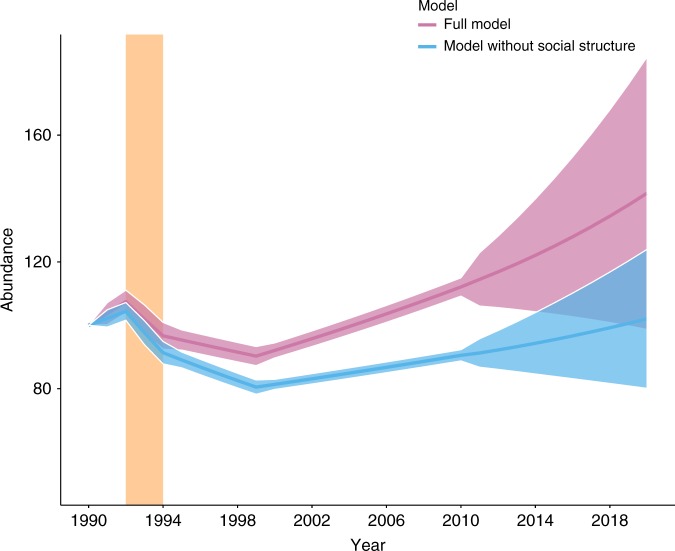
Mean abundance of female hyenas. Mean abundance (±95% confidence intervals) of female hyenas projected throughout the study period (1990–2010) and predicted beyond (2010–2020) based on the full model (pink) and a model without social structure (blue). The vertical bar (light orange) represents the period (1993–1994) during which the CDV epidemic occurred. This figure does not illustrate the actual number of hyenas in the study population; the population growth rate estimates from each period were used to produce it. The starting abundance was indexed as 100

We projected changes in population size using the values of *λ* obtained for each of the three periods onto the period from 2000 and 2010, when the virulent strain was absent from the ecosystem (Fig. 5, pink curve—see Supplementary Fig. 7 and Supplementary Results for an alternative model). In addition, we predicted changes in population size between 2010 and 2020, based on the parameters estimated for the period 2000–2010. Ecological resistance, the opposite of the magnitude of the reduction (16%) in population size, was 84% and recovery time, the time needed for the population to regain its pre-epidemic size, was 16 years.

We fitted a MECMR model without any contribution of social status to all processes to show how population size would vary between 1990 and 2020 if all parameter values would be similar for high-ranking and low-ranking females (Fig. 5, blue curve). By doing so, we highlight the role of high-ranking females in increasing ecological resistance and accelerating population recovery after the epidemic. For this model, the drop in population size was 23%, ecological resistance was therefore 77 % and full recovery was not reached by 2020 (Fig. 5).

## Discussion

The predicted impact of the CDV epidemic on female hyenas was severe. The projected hyena population was reduced by 16% and needed more than a decade to return to its pre-epidemic size. CDV is a global multi-host pathogen, infecting and causing mortality in a wide range of hosts, mostly carnivores^23^. In Africa, CDV is considered to be an exotic pathogen imported from the USA to South Africa by human activity in the previous century^24^. As expected from other morbilliviruses, such as measles or phocine distemper virus, *R*_0_ in hyenas was elevated during the epidemic, demonstrating the capacity of the novel, virulent strain first observed during this epidemic to invade its new host population in 1993. Here we estimated a CDV *R*_0_ in a closed hyena population, not considering contacts between hyenas and lions or other carnivores present in the Serengeti ecosystem. We consider it unlikely that our estimate of CDV *R*_0_ in hyenas was substantially affected by other carnivores since CDV transmission occurs primarily via physical contacts, the frequency of contacts among hyenas (which are highly social) is substantially higher than between hyenas and other carnivores, and infectiousness was mostly among juveniles stationed at communal dens, who have limited contact to other non-canid species shedding CDV^20,25^. Adults allocated to the infected state were likely not infectious themselves, i.e. not actively shedding the virus^10,19,20^. Nikolin et al.^19^ provide a more detailed discussion of the likely involvement of various carnivore species from an ecosystem-wide perspective.

An interesting finding was that reproductive values varied little with infection states (Supplementary Fig. 6). This is probably because subclinical CDV infection in adult females, if present, did not reduce their survival or chance of becoming a breeder (see Table 1 and ref. ^10^), and possibly also because of the rapid transition to the recovered state. Had infection negatively impacted female survival or fecundity, we would have expected larger differences in female reproductive values between the susceptible, infected and recovered states. Interestingly, CDV *R*_0_ decreased as the probability for high-ranking females to maintain their social status increased, probably because high-ranking females were more likely to produce a litter than low-ranking females, and their cubs were less likely to become infected and to die of CDV infection (Table 1). We detected an elevated demographic contribution by high-ranking females to population growth (Fig. 2). The importance of high social status for population growth is further illustrated by the predicted persistent population reduction and lack of full recovery when we modelled the female population as homogeneous in terms of social states (Fig. 5, blue curve).

In contrast to the lion population, which was reduced by 30% during the same epidemic^18^, hyenas were relatively ecologically resistant with a potential reduction of only 16%. This difference could be explained by the fact that in hyenas CDV infection only decreased juvenile survival (Table 1), whereas in lions mortality occurred in all age classes^18^. As the hyena population growth rate was highly sensitive to adult survival (Fig. 2), the ecological resistance of hyenas would have been far lower if infection had decreased adult survival.

Although survival of adult hyenas was not affected by CDV, our projections show that hyenas needed potentially more time to recover from this epidemic (~16 years, Fig. 5) than lions (~3 years)^26^. The most likely reasons for such a difference between the two species are different life histories and maternal investment patterns. Lionesses have no dominance hierarchy, produce litters of 1–6 cubs, nurse communally and share food^27^. In contrast, female hyenas have a linear dominance hierarchy and compete intensively for food, produce smaller litters of 1–3 cubs, and do not nurse communally. Instead, hyenas provide exceptionally rich milk and high maternal input in terms of individual lactation effort which can last for 12–20 months^28,29^ and intense within-litter competition can result in a reduction in litter size^30^. During the epidemic, hyena cub mortality was also spread across several cohorts born between 1993 and 1994, increasing the time required for the population to compensate increased cub mortality. Our results thus suggest that for *K*-selected species with a slow development of young, high resilience may depend on high ecological resistance to the initial epidemic rather than a fast recovery if pathogen infection is virulent to juveniles but benign to adults. In addition, in social species with dominance hierarchies, it is likely that high-ranking animals will substantially contribute to population recovery after an outbreak.

Epidemics of emerging infectious diseases pose a threat to many *K*-selected species. For example, Ebola outbreaks recently caused a severe decline in a western lowland gorilla population; this population has yet to recover^11^. Substantial advances in our understanding of infectious diseases and ability to forecast their outcome has been considerably improved by the development of continuous time models^31,32^. However, the theoretical development of these models has outpaced their fusion with data^33^, partly owing to the practical difficulty of estimating their parameters^34^. To accurately predict and improve the diagnosis of the impact of future epidemics on vulnerable wildlife populations, it is thus essential that we develop appropriate data-driven models. Because discrete time modelling approaches can easily incorporate (typically uncertain) field data and because they allow the interactions between host demography, sociality and disease dynamics to be assessed, we expect their use to increase in disease ecology. By monitoring changes in the stable state proportions of individuals in terms of susceptible, infected and recovered (Supplementary Fig. 3) through time, discrete time modelling approaches allow to predict when a population is at risk following the emergence of a virulent strain. This would be expected when a higher proportion of susceptible individuals than recovered ones occurs. These types of models can also be useful to compare the outcomes of management interventions by predicting their impacts on changes in population size and assessing which vital rates have the greatest impact on population viability^14,33^. One interesting development of our deterministic model, which assumes constant environmental effects and constant survival rates during each epidemic period, would be to include the effects of stochastic forces (environment or demography) or density-dependent or frequency-dependent processes on the dynamics of disease transmission^14,15^.

## Methods

### MECMR model

In a previous study, we developed and validated a MECMR model to quantify yearly survival and transition probabilities between demographic, social and infection states in three hyena clans monitored for two decades in the Serengeti National Park that were infected with CDV during the disease epidemic of 1993/1994^10^. Here, we adapted this MECMR model to include temporal effects on survival and infection probabilities, and used the parameter estimates of this MECMR model (Table 1) as input for the stage-structured matrix population model. We present all methods and sample sizes established in our previous study^10^ in detail in the Supplementary Methods. In the next section, we explain how we assigned individual hyenas to their demographic, social and infection state, as the states used in the MECMR model match with those used in the matrix population model in the current study. All procedures in our previous study^10^ were performed in accordance with the requirements of the Tanzanian authorities who issued appropriate research permits (1990-xxx-ER-90-130 to 2018-321-NA-90-130) and The Ethics Committee on Animal Welfare at the Leibniz Institute for Zoo and Wildlife Research, Berlin, Germany (permit number: 2014-09-03).

### The states of the matrix population model

We only modelled the female section of the hyena population, as typical for demography models, which often focus on the philopatric sex^22^. The four demographic states for female hyenas in the stage-structured matrix population model were as follows: cub (C), subadult (SA), breeder (B) and non-breeder (NB) (Table 2). Cubs were younger than 1 year, subadults aged between 1 and 2 years. Cubs entirely depended on maternal milk for at least the first 6 months, subadults at least partially, as weaning occurs at 12–20 months of age^28,29^. Breeders gave birth to a litter during a given year, as documented by a freshly ruptured clitoris caused by parturition^35^ and/or subsequent lactation, whereas non-breeders did not. As breeders were mostly lactating females, considering this state allowed us to account for the elevated energetic cost of lactation and the possible delayed and indirect costs of females losing their offspring during the outbreak^30^.

**Table 2:** Notation and definition of demographic, social and infection states in the stage-structured matrix population model

The 4 demographic states	The 2 social states	The 3 infection states
Cub (C): 0 − 365 days	Low social status (L): below median standardised social rank (low-ranking)	Susceptible (S): never infected with CDV
Subadult (SA): 366 − 720 days		Infected (I): infected with CDV
Adult breeder (B): have given birth to a litter (that year)	High social status (H): equal or above median standardised social rank (high-ranking)	Recovered (R): immune to CDV
Adult non-breeder (NB): have not given birth to a litter (that year)		

These states were assigned to 625 female hyenas each year during 1990 and 2010. We considered uncertainty in the assignment of infection states. For further details on the methods used to assign these states see^10^ and Supplementary Methods

The two social states were low (L) or high social status (H) (Table 2). These social states were based on the positions of females in strictly linear adult female dominance hierarchies in study clans. To construct these hierarchies we recorded, for each clan, submissive behaviours during dyadic interactions between adult females^30,36–38^. Hierarchies were updated after recruitment or loss of adult females and after periods of social instability. To compare the social status of each female (across clans and years), females were assigned standardised ranks^30,36–38^. Standardised ranks were evenly distributed between the highest (+1) and lowest (−1) standardised rank. Adult (i.e. breeder and non-breeder) females were classified as holding a high status if their standardised rank was between 0.01 and +1 or a low status if their standardised rank was between −1 and 0.0^10^. Every year, we assigned half of the females with a standardised rank above the median to the high social status H and half of the female with a standardised rank below the median standardised rank to low social status L^10^. We assigned the social state of the genetic mother to non-adopted cubs and subadults, and that of the surrogate mother to adopted ones^10^ (as offspring typically acquire a rank immediately below that of the female that reared them^38^). High-ranking females have preferential access to food resources in clan territories and commute less frequently to forage in areas outside the territory than low-status females. As a result, they produce their first litter at an earlier age^37^ and produce more milk, which results in their cubs growing faster and surviving better to adulthood than the cubs of low-ranking females^37^. We found previously that the cubs of high status females have a higher probability of surviving infection with CDV (Table 1) than those of low-ranking females^10^.

The three infection states for female hyenas were susceptible (S), infected (I) and recovered (R) (Table 2). The outcomes of diagnostic procedures were employed to assign these states; (1) RT-PCR screening for the presence or absence of CDV RNA in samples, (2) CDV antibody titres in serum and (3) the observation of clinical signs associated with CDV infection in hyenas, and the secondary infections it causes in this species^20^ (for more details on these procedures, including on antibodies used and sample sizes, see the Supplementary Methods and Supplementary Table 1). CDV infection could occur only once in life because hyenas develop life-long immunity to this virus if they survive the infection^10,23^. As a result, any individual hyena classified as susceptible in a given year was further classified as susceptible during all previous years when the individual was detected; infected in a given year was classified as susceptible during all previous years, and recovered during all subsequent years following the infected state*;* recovered in a given year was classified as recovered during all subsequent years when the individual was detected^10^.

SIR models generally assume that (all) infected animals are infectious, but this is not the case for CDV^39^ and some other pathogens such as herpes viruses^40^. Animals infected with CDV start shedding virus (i.e., become infectious) when epithelial cells are infected, which marks the onset of clinical Distemper^39^. The initial stage of CDV infection in blood immune cells induces the production of antibodies against CDV, but at this stage virus is not shed and infection can be cleared before Distemper develops^23^. As a result, not all hyenas infected with CDV were infectious and Distemper was overwhelmingly observed in young hyenas^10,19,20^.

Our MECMR model^10^ included individuals with an unknown infection state, that is, we modelled uncertainty on the infection state (Supplementary Methods). As we focused on uncertainty related to infection states, we did not include a small fraction of individuals with unknown or unclear demographic and social states. Including such individuals would imply to account also for uncertainty on the demographic and social states and this would result in over-parameterised MECMR models. Excluding this small fraction of individuals is unlikely to have biased the parameters estimated in this study because MECMR models account for potential heterogeneity in detection probability^16^ and sample sizes remained large even after exclusion of this fraction^10^. All females were highly detectable, regardless of their demographic or social state, as we show in^10^ and in the Supplementary Results. To fulfil a main assumption of CMR analyses on sampling design, we focused exclusively on hyenas detected during our routine observations at clan communal dens, i.e. did not include hyenas that were detected opportunistically away from clan communal den areas.

As we were interested in the key impact and the long-term perspective (20 years) of the impact of the disease on population dynamics rather than a fine-scaled, short-term one, the duration of the infected state in our model exceeded the actual duration of the infectious period with CDV^20^. For the same reason, we did not include an exposed state, i.e., we did not model SEIR dynamics.

CDV prevalence and the proportion of infected females in each demographic state are shown in Supplementary Fig. 8 and Supplementary Fig. 9, respectively. Sample sizes for each combination of a given demographic, social and infection state are provided in the Supplementary Table 2.

### Structure of the meta-matrix

The overall stage-structured matrix population model, termed the meta-matrix **M**, was of the form:

1 $$\begin{array}{l}\begin{array}{*{20}{l}} \quad \hfill & \quad \hfill & {\;\,{\mathrm{C}}} \hfill & {\quad {\mathrm{SA}}} \hfill & {\quad {\mathrm{B}}} \hfill & {\quad {\mathrm{NB}}} \hfill & \, \hfill \end{array}\\ {\mathbf{M}} = \begin{array}{*{20}{c}} {\mathrm{C}} \\ {{\mathrm{SA}}} \\ {\mathrm{B}} \\ {{\mathrm{NB}}} \end{array}\left[ {\begin{array}{*{20}{c}} {\mathbf{Z}} & {\mathbf{Z}} & {\mathbf{F}} & {\mathbf{Z}} \\ {{{\mathbf{SA}}_{\mathrm{C}}}} & {\mathbf{Z}} & {\mathbf{Z}} & {\mathbf{Z}} \\ {\mathbf{Z}} & {{{\mathbf{B}}_{\mathrm{SA}}}} & {{\mathbf{B}}_{\mathrm{B}}} & {{{\mathbf{B}}_{\mathrm{NB}}}} \\ {\mathbf{Z}} & {{{\mathbf{NB}}_{\mathrm{SA}}}} & {{{\mathbf{NB}}_{\mathrm{B}}}} & {{{\mathbf{NB}}_{\mathrm{NB}}}} \end{array}} \right]\end{array}$$

Each entry in **M** in Eq. (1) was a 6×6 submatrix that accounted for survival, social and infection processes also corresponding to the transition from a starting demographic state (4 columns corresponding to the demographic state: cub (C), subadult (SA), breeder (B), non-breeder (NB) on the top of each column in Eq. (1)) to the following demographic state (4 rows corresponding to C, SA, B, NB, on the left side of the matrix). Thus, **M** presented in Eq. (1) is a block-matrix notation of total dimension 24×24. By combining the demographic, social and infection states, the female part of the population at time *t* was represented by a vector **n**(*t*) with 4 × 2 × 3 = 24 components.

Using the notation from Table 2, the 24 state combinations ranged from Cub—Low social status—Susceptible to Non-breeder—High social status—Recovered, i.e. the infection state varied within the social state, itself varying within the demographic state, in the sequence detailed in Table 2. We used a discrete one-year projection interval, as input parameter estimates were calculated on a yearly basis^10^. The 24×24 meta-matrix **M** linked **n**(*t*) to **n**(*t* + 1) as:

2 $${\mathbf{n}}\left( {t + 1} \right) = {\mathbf{M}} \times {\mathbf{n}}\left( t \right)$$

**Fig. 6:**
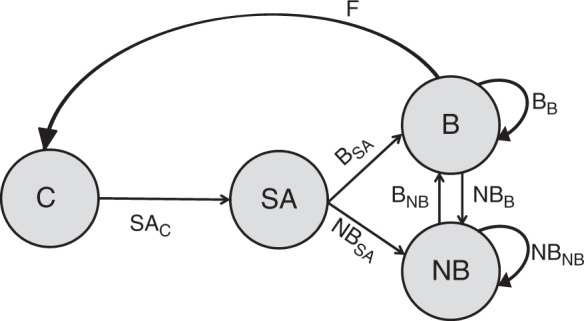
Population model. Structure of the overall matrix population model or meta-matrix **M** in Eq. (1), with its 8 survival-transition submatrices indicated in bold. Arrows represent the survival-transition probabilities of female hyenas, i.e. the transitions between the 4 demographic states (shown as grey circles and with C cub, SA subadult, B breeder, NB non-breeder), conditional on survival and considering potential changes in social and infection states. The matrices were named according to the following demographic state, with the starting demographic state as index. **F** was the fertility matrix, accounting for cub production by breeders

The notation **Z** in Eq. (1) was for a null 6×6 submatrix, indicating that there was no transition from the 6 columns corresponding to the 6 social × infection states in this demographic state to the corresponding 6 rows in this demographic state. For instance, the first entry in **M** (Eq. (1), top left) is a null 6×6 submatrix because there was no transition from the cub state to the cub state, as by definition all surviving cubs became subadults (Table 2). The notations **SA**_C_, **B**_SA_, **NB**_SA_, **F**, **B**_B_, **NB**_B_, **B**_NB_ and **NB**_NB_ in Eq. (1) corresponded to 6×6 survival-transition submatrices, named according to the following demographic state, with the starting demographic state as index, using the state notations from Table 2. These submatrices accounted for demographic transitions for female hyenas, conditional on their survival and considering potential changes in social states and in infection states. For instance, **SA**_C_ represented the survival-transition probabilities from the cub state to the subadult state. The notation **F** in Eq. (1) corresponded to a 6×6 submatrix that modelled fertility. The overall matrix population model **M** can also be represented as a life cycle graph (Fig. 6).

### Assembling the submatrices

All processes (transitions between states and survival) were first modelled separately from each other and then combined in a way described here. The 6×6 submatrices in **M** in Eq. (1) were obtained by appropriate products of parameters in separate matrices (described in the next section), to generate successive events within a 1 year projection interval: change in demographic state, change in social state, change in infection state and then survival. For instance, the survival-transition submatrix to the breeder state from the subadult state (**B**_SA_ in Eq. (1), Fig. 6) was expressed as:

3 $${\mathbf{B}}_{{\mathrm{SA}}} = {\mathbf{Survival}}_{\mathrm{B}} \times {\mathbf{Infection}} \times {\mathbf{Social}} \times {\mathbf{DemoB}}_{{\mathrm{SA}}}$$

In Eq. (3), **Survival**_B_ was a 6×6 submatrix accounting for the survival of breeders, **Infection** a 6×6 submatrix accounting for transitions among infection states, **Social** a 6×6 submatrix accounting for transitions among social states and **DemoB**_SA_ a 6×6 submatrix accounting for the demographic transitions, i.e. the probability of becoming a breeder (recruitment) for subadults.

The other submatrices **SA**_C_**, NB**_**SA**_**, B**_B_**, NB**_B_ and **NB**_NB_ in **M** (Eq. (1), Fig. 6) were built using the same logic, and were as follows:

4 $${\mathbf{SA}}_{\mathrm{C}} = {\mathbf{Survival}}_{{\mathrm{SA}}} \times {\mathbf{Infection}}$$

5 $${\mathbf{NB}}_{{\mathrm{SA}}} = {\mathbf{Survival}}_{{\mathrm{NB}}} \times {\mathbf{Infection}} \times {\mathbf{Social}} \times {\mathbf{DemoNB}}_{{\mathrm{SA}}}$$

6 $${\mathbf{B}}_{\mathrm{B}} = {\mathbf{Survival}}_{\mathrm{B}} \times {\mathbf{Infection}} \times {\mathbf{Social}} \times {\mathbf{DemoB}}_{\mathrm{B}}$$

7 $${\mathbf{NB}}_{\mathrm{B}} = {\mathbf{Survival}}_{{\mathrm{NB}}} \times {\mathbf{Infection}} \times {\mathbf{Social}} \times {\mathbf{DemoNB}}_{\mathrm{B}}$$

8 $${\mathbf{B}}_{{\mathrm{NB}}} = {\mathbf{Survival}}_{\mathrm{B}} \times {\mathbf{Infection}} \times {\mathbf{Social}} \times {\mathbf{DemoB}}_{{\mathrm{NB}}}$$

9 $${\mathbf{NB}}_{{\mathrm{NB}}} = {\mathbf{Survival}}_{{\mathrm{NB}}} \times {\mathbf{Infection}} \times {\mathbf{Social}} \times {\mathbf{DemoNB}}_{{\mathrm{NB}}}$$

For the 6×6 submatrix accounting for fertility **F** (Eq. (1), Fig. 6), we assumed that all births and deaths occurred simultaneously at the end of the projection interval, that is, we modelled the population with a birth pulse reproduction and a pre-breeding census^22^. Heterogeneity in the timing of births was handled in the statistical analysis of our data. The fertility submatrix was:

10 $${\mathbf{F}} = {\mathbf{Survival}}_{\mathrm{C}} \times {\mathbf{Infection}}_{\mathrm{C}} \times {\mathbf{Infection}}_{\mathrm{N}} \times {\mathbf{fecundity}}$$

In Eq. (10), **Survival**_C_ was a 6×6 submatrix accounting for cub survival, **Infection**_C_ a 6×6 submatrix accounting for transitions between infection states for cubs, **Infection**_N_ a 6×6 submatrix accounting for transitions between infection states for newborns and **fecundity**, the elementary fecundity submatrix.

The structure of such block-matrix model in **M** (Eq. (1)) was similar to the structure of the MECMR model used to estimate demographic, social and infection parameters, reflecting the transitions from one state to another on a yearly basis^10^.

### Description of all submatrices

Each entry in the submatrices described below corresponded to the probability of transition (or survival), or the fecundity, from a starting combination of social and infection states to a following combination of social and infection states. As indicated above, the 6 rows and 6 columns in these submatrices corresponded to: Low social status—Susceptible, Low social status—Infected, Low social status—Recovered, High social status—Susceptible, High social status—Infected, High social status—Recovered.

**Fig. 7:**
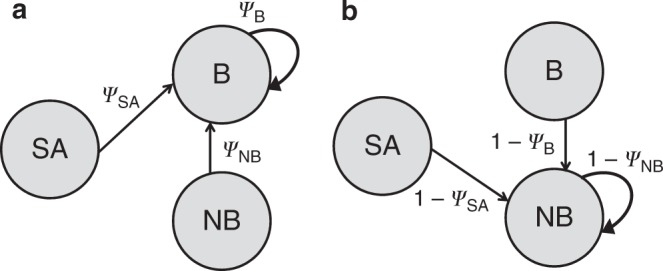
Transitions between demographic states. Transition to **a** the breeder state and **b** the non-breeder state, for female hyenas. The grey circles show the 3 demographic states from which the breeder and non-breeder states can be accessed (SA subadult, B breeder, NB non-breeder). Arrows and symbols in italics represent the transition probabilities to **a** the breeder state from subadults (*ψ*_SA_), breeders (*ψ*_B_) and non-breeders (*ψ*_NB_) and **b** to the non-breeder state from subadults (1−*ψ*_SA_), breeders (1−*ψ*_B_) and non-breeders (1−*ψ*_NB_). All transition probabilities varied with social status (see Table 1 for parameter notations and estimates) − this is not shown, for simplicity

We built six diagonal demography submatrices; three to account for the transition to the breeder state (**DemoB**_SA_, **DemoB**_B_, **DemoB**_NB_) (Fig. 7a) and three others to account for the transition to the non-breeder state (**DemoNB**_SA_, **DemoNB**_B_, **DemoNB**_NB_) (Fig. 7b), both accessed from the subadult, breeder or non-breeder state. The parameter *ψ* in these submatrices denoted the transition probability to the breeder state and 1−*ψ* the transition probability to the non-breeder state. As indicated above and in Table 2, all surviving cubs became subadults by definition, explaining why there is no demography submatrix in Eq. (4). These matrices were as follows:

11 $${{\mathbf{DemoB}}_{\mathrm{SA}}} = \begin{array}{*{20}{c}} {{\mathrm{L}} - {\mathrm{S}}} \\ {{\mathrm{L}} - {\mathrm{I}}} \\ {{\mathrm{L}} - {\mathrm{R}}} \\ {{\mathrm{H}} - {\mathrm{S}}} \\ {{\mathrm{H}} - {\mathrm{I}}} \\ {{\mathrm{H}} - {\mathrm{R}}} \end{array}\left[ {\begin{array}{*{20}{c}} {\psi {\mathrm{SAL}}} & 0 & 0 & 0 & 0 & 0 \\ 0 & {\psi {\mathrm{SAL}}} & 0 & 0 & 0 & 0 \\ 0 & 0 & {\psi {\mathrm{SAL}}} & 0 & 0 & 0 \\ 0 & 0 & 0 & {\psi {\mathrm{SAH}}} & 0 & 0 \\ 0 & 0 & 0 & 0 & {\psi {\mathrm{SAH}}} & 0 \\ 0 & 0 & 0 & 0 & 0 & {\psi {\mathrm{SAH}}} \end{array}} \right]$$

12 $${{\mathbf{DemoB}}_{\mathrm{B}}} = \begin{array}{*{20}{c}} {{\mathrm{L}} - {\mathrm{S}}} \\ {{\mathrm{L}} - {\mathrm{I}}} \\ {{\mathrm{L}} - {\mathrm{R}}} \\ {{\mathrm{H}} - {\mathrm{S}}} \\ {{\mathrm{H}} - {\mathrm{I}}} \\ {{\mathrm{H}} - {\mathrm{R}}} \end{array}\left[ {\begin{array}{*{20}{c}} {\psi {\mathrm{BL}}} & 0 & 0 & 0 & 0 & 0 \\ 0 & {\psi {\mathrm{BL}}} & 0 & 0 & 0 & 0 \\ 0 & 0 & {\psi {\mathrm{BL}}} & 0 & 0 & 0 \\ 0 & 0 & 0 & {\psi {\mathrm{BH}}} & 0 & 0 \\ 0 & 0 & 0 & 0 & {\psi {\mathrm{BH}}} & 0 \\ 0 & 0 & 0 & 0 & 0 & {\psi {\mathrm{BH}}} \end{array}} \right]$$

13 $${{\mathbf{DemoB}}_{\mathrm{NB}}} = \begin{array}{*{20}{c}} {{\mathrm{L}} - {\mathrm{S}}} \\ {{\mathrm{L}} - {\mathrm{I}}} \\ {{\mathrm{L}} - {\mathrm{R}}} \\ {{\mathrm{H}} - {\mathrm{S}}} \\ {{\mathrm{H}} - {\mathrm{I}}} \\ {{\mathrm{H}} - {\mathrm{R}}} \end{array}\left[ {\begin{array}{*{20}{c}} {\psi {\mathrm{NBL}}} & 0 & 0 & 0 & 0 & 0 \\ 0 & {\psi {\mathrm{NBL}}} & 0 & 0 & 0 & 0 \\ 0 & 0 & {\psi {\mathrm{NBL}}} & 0 & 0 & 0 \\ 0 & 0 & 0 & {\psi {\mathrm{NBH}}} & 0 & 0 \\ 0 & 0 & 0 & 0 & {\psi {\mathrm{NBH}}} & 0 \\ 0 & 0 & 0 & 0 & 0 & {\psi {\mathrm{NBH}}} \end{array}} \right]$$

14 $${{{\mathbf{DemoNB}}_{\mathrm{SA}}} = \begin{array}{*{20}{c}} {{\mathrm{L}} - {\mathrm{S}}} \\ {{\mathrm{L}} - {\mathrm{I}}} \\ {{\mathrm{L}} - {\mathrm{R}}} \\ {{\mathrm{H}} - {\mathrm{S}}} \\ {{\mathrm{H}} - {\mathrm{I}}} \\ {{\mathrm{H}} - {\mathrm{R}}} \end{array}\left[ {\begin{array}{*{20}{c}} {1 - \psi {\mathrm{SAL}}} & 0 & 0 & 0 & 0 & 0 \\ 0 & {1 - \psi {\mathrm{SAL}}} & 0 & 0 & 0 & 0 \\ 0 & 0 & {1 - \psi {\mathrm{SAL}}} & 0 & 0 & 0 \\ 0 & 0 & 0 & {1 - \psi {\mathrm{SAH}}} & 0 & 0 \\ 0 & 0 & 0 & 0 & {1 - \psi {\mathrm{SAH}}} & 0 \\ 0 & 0 & 0 & 0 & 0 & {1 - \psi {\mathrm{SAH}}} \end{array}} \right]}$$

15 $${{{\mathbf{DemoNB}}_{\mathrm{B}}} = \begin{array}{*{20}{c}} {{\mathrm{L}} - {\mathrm{S}}} \\ {{\mathrm{L}} - {\mathrm{I}}} \\ {{\mathrm{L}} - {\mathrm{R}}} \\ {{\mathrm{H}} - {\mathrm{S}}} \\ {{\mathrm{H}} - {\mathrm{I}}} \\ {{\mathrm{H}} - {\mathrm{R}}} \end{array}\left[ {\begin{array}{*{20}{c}} {1 - \psi {\mathrm{BL}}} & 0 & 0 & 0 & 0 & 0 \\ 0 & {1 - \psi {\mathrm{BL}}} & 0 & 0 & 0 & 0 \\ 0 & 0 & {1 - \psi {\mathrm{BL}}} & 0 & 0 & 0 \\ 0 & 0 & 0 & {1 - \psi {\mathrm{BH}}} & 0 & 0 \\ 0 & 0 & 0 & 0 & {1 - \psi {\mathrm{BH}}} & 0 \\ 0 & 0 & 0 & 0 & 0 & {1 - \psi {\mathrm{BH}}} \end{array}} \right]}$$

16 $${{{\mathbf{DemoNB}}_{\mathrm{NB}}} = \begin{array}{*{20}{c}} {{\mathrm{L}} - {\mathrm{S}}} \\ {{\mathrm{L}} - {\mathrm{I}}} \\ {{\mathrm{L}} - {\mathrm{R}}} \\ {{\mathrm{H}} - {\mathrm{S}}} \\ {{\mathrm{H}} - {\mathrm{I}}} \\ {{\mathrm{H}} - {\mathrm{R}}} \end{array}\left[ {\begin{array}{*{20}{c}} {1 - \psi {\mathrm{NBL}}} & 0 & 0 & 0 & 0 & 0 \\ 0 & {1 - \psi {\mathrm{NBL}}} & 0 & 0 & 0 & 0 \\ 0 & 0 & {1 - \psi {\mathrm{NBL}}} & 0 & 0 & 0 \\ 0 & 0 & 0 & {1 - \psi {\mathrm{NBH}}} & 0 & 0 \\ 0 & 0 & 0 & 0 & {1 - \psi {\mathrm{NBH}}} & 0 \\ 0 & 0 & 0 & 0 & 0 & {1 - \psi {\mathrm{NBH}}} \end{array}} \right]}$$

**Fig. 8:**
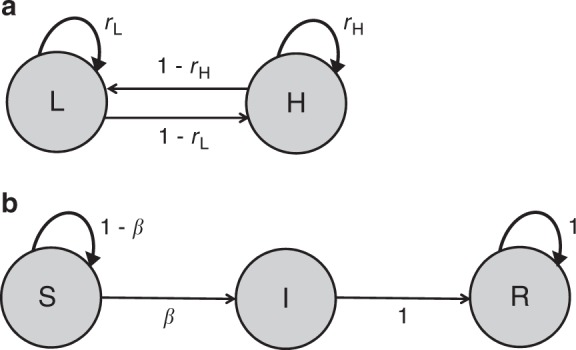
Transitions between social and infection states for female hyenas. **a** Transitions between social states in subadult, breeder and non-breeder female hyenas corresponded to the submatrix **Social** (Eq. (17)). The two social states are indicated as grey circles (L: low ranking; H: high-ranking). The arrows show the transition probabilities, with *r*_L_ and *r*_H_ the probabilities of staying in a low or high social state, respectively, and with 1−*r*_L_ and 1−*r*_H_ the probabilities of becoming high or low-ranking, respectively. **b** Transitions between infection states in subadult, breeder and non-breeder female hyenas, corresponding to the submatrix **Infection** (Eq. (20)). The three infection states are indicated as grey circles (S susceptible, I infected, R recovered). The arrows show the transition probability between those states, with *β* the probability of transition from a susceptible state to an infected state (the infection probability). The infection probability varied with the social state (see Table 1) − this is not shown, for simplicity

We built a social submatrix to account for the fact that subadults, breeders and non-breeders could either stay within their social state or change it (Fig. 8a). The parameters *r*_L_ and *r*_H_ in this submatrix represented the probabilities of staying in a low or high social state, respectively, and 1−*r*_L_ and 1−*r*_H_ the probabilities of becoming high or low ranking, respectively. Cubs had the same social state as their genetic or surrogate mother^38^; which explains why this social submatrix does not appear in Eq. (4). This submatrix was as follows:

17 $${\mathbf{Social}} = \begin{array}{*{20}{c}} {{\mathrm{L}} - {\mathrm{S}}} \\ {{\mathrm{L}} - {\mathrm{I}}} \\ {{\mathrm{L}} - {\mathrm{R}}} \\ {{\mathrm{H}} - {\mathrm{S}}} \\ {{\mathrm{H}} - {\mathrm{I}}} \\ {{\mathrm{H}} - {\mathrm{R}}} \end{array}\left[ {\begin{array}{*{20}{c}} {r{\mathrm{L}}} & 0 & 0 & {1 - r{\mathrm{H}}} & 0 & 0 \\ 0 & {r{\mathrm{L}}} & 0 & 0 & {1 - r{\mathrm{H}}} & 0 \\ 0 & 0 & {r{\mathrm{L}}} & 0 & 0 & {1 - r{\mathrm{H}}} \\ {1 - r{\mathrm{L}}} & 0 & 0 & {r{\mathrm{H}}} & 0 & 0 \\ 0 & {1 - r{\mathrm{L}}} & 0 & 0 & {r{\mathrm{H}}} & 0 \\ 0 & 0 & {1 - r{\mathrm{L}}} & 0 & 0 & {r{\mathrm{H}}} \end{array}} \right]$$

We built three infection submatrices: one for newborns (**Infection**_N_, Eq. (18)), one for cubs (**Infection**_C_, Eq. (19)) and one for subadults, breeders and non-breeders (**Infection**, Eq. (20), Fig. 8b). As mentioned above, we considered that infected individuals became recovered at the following interval, conditional on their survival. We also considered that recovered individuals acquired life-long immunity to CDV, which implied that they remained recovered until their death or disappearance (as in^10^). For the infection submatrix for newborns **Infection**_N_, part of the submatrix **F** (in Eqs. (1) and (10), Fig. 6), we considered that mothers only produced susceptible newborns, irrespective of their own infection status. Indeed, as we assumed that adults were not infectious and hence did not excrete the virus actively as young individuals^10,19,20^, we considered the transmission of CDV to be strictly horizontal. These key features of the model generated susceptible individuals in the model population at each projection interval (in addition to those already present in the initial population vector, details below). The 6 × 6 matrix for newborns, **Infection**_N_, was as follows:

18 $${{\mathbf{Infection}}_{\mathrm{N}}} = \begin{array}{*{20}{c}} {{\mathrm{L}} - {\mathrm{S}}} \\ {{\mathrm{L}} - {\mathrm{I}}} \\ {{\mathrm{L}} - {\mathrm{R}}} \\ {{\mathrm{H}} - {\mathrm{S}}} \\ {{\mathrm{H}} - {\mathrm{I}}} \\ {{\mathrm{H}} - {\mathrm{R}}} \end{array}\left[ {\begin{array}{*{20}{c}} 1 & 1 & 1 & 0 & 0 & 0 \\ 0 & 0 & 0 & 0 & 0 & 0 \\ 0 & 0 & 0 & 0 & 0 & 0 \\ 0 & 0 & 0 & 1 & 1 & 1 \\ 0 & 0 & 0 & 0 & 0 & 0 \\ 0 & 0 & 0 & 0 & 0 & 0 \end{array}} \right]$$

In Eq. (18), the value 1 represented the production of susceptible newborns by mothers of low social status and high social status (by definition all breeders produced a litter thus the probability of female offspring production was equal to 1).

For cubs, the only possible infection transitions were from a susceptible to a susceptible state or from a susceptible to an infected state. The submatrix for cubs, **Infection**_C_ (Eq. (19)) was thus:

19 $${{\mathbf{Infection}}_{\mathrm{C}}} = \begin{array}{*{20}{c}} {{\mathrm{L}} - {\mathrm{S}}} \\ {{\mathrm{L}} - {\mathrm{I}}} \\ {{\mathrm{L}} - {\mathrm{R}}} \\ {{\mathrm{H}} - {\mathrm{S}}} \\ {{\mathrm{H}} - {\mathrm{I}}} \\ {{\mathrm{H}} - {\mathrm{R}}} \end{array}\left[ {\begin{array}{*{20}{c}} {1 - \beta {\mathrm{CL}}} & 0 & 0 & 0 & 0 & 0 \\ {\beta {\mathrm{CL}}} & 0 & 0 & 0 & 0 & 0 \\ 0 & 1 & 0 & 0 & 0 & 0 \\ 0 & 0 & 0 & {1 - \beta {\mathrm{CH}}} & 0 & 0 \\ 0 & 0 & 0 & {\beta {\mathrm{CH}}} & 0 & 0 \\ 0 & 0 & 0 & 0 & 1 & 0 \end{array}} \right]$$

In Eq. (19), *β*_CL_ and *β*_CH_ were the infection probabilities for low-ranking and high-ranking cubs, respectively, i.e. the probabilities of transition from a susceptible to an infected state (and hence 1−*β*_CL_ and 1−*β*_CH_ the probabilities of transition from a susceptible to a susceptible state for low-ranking and high-ranking cubs, respectively). The values 1 corresponded to infected cubs becoming recovered at the next interval (conditional on their survival). As there was no cub in the recovered state in the data set^10^, there was no transition from recovered cub to recovered cub.

The submatrix **Infection** accounted for possible transitions between susceptible, infected and recovered states for subadults, breeders and non-breeders (Fig. 8b). *β*_L_ and *β*_H_ were the infection probabilities for low-ranking and high-ranking subadults, breeders and non-breeders, respectively, i.e. the probabilities of transition from a susceptible to an infected state (and hence 1−*β*_L_ and 1−*β*_H_ the probabilities of transition from a susceptible to a susceptible state for low-ranking and high-ranking subadults, breeders and non-breeders, respectively). The values *1* in corresponded to infected individuals becoming or staying recovered at the next interval (conditional on their survival) (Fig. 8b). This matrix was as follows:

20 $${\mathbf{Infection}} = \begin{array}{*{20}{c}} {{\mathrm{L}} - {\mathrm{S}}} \\ {{\mathrm{L}} - {\mathrm{I}}} \\ {{\mathrm{L}} - {\mathrm{R}}} \\ {{\mathrm{H}} - {\mathrm{S}}} \\ {{\mathrm{H}} - {\mathrm{I}}} \\ {{\mathrm{H}} - {\mathrm{R}}} \end{array}\left[ {\begin{array}{*{20}{c}} {1 - \beta {\mathrm{L}}} & 0 & 0 & 0 & 0 & 0 \\ {\beta {\mathrm{L}}} & 0 & 0 & 0 & 0 & 0 \\ 0 & 1 & 1 & 0 & 0 & 0 \\ 0 & 0 & 0 & {1 - \beta {\mathrm{H}}} & 0 & 0 \\ 0 & 0 & 0 & {\beta {\mathrm{H}}} & 0 & 0 \\ 0 & 0 & 0 & 0 & 1 & 1 \end{array}} \right]$$

In accordance with the notation in the matrix **M** (Eq. (1), also see Fig. 6), young females entered the population as cubs, born from breeders. As all females in the breeder state reproduced by definition (Table 2), the fecundity value was set to 1. Consequently, the elementary fecundity submatrix of newborn females per breeder female (then combined with the other submatrices in the fertility matrix) was as follows:

21 $${\mathbf{fecundity}} = sex.ratio \times litter.size \times \begin{array}{*{20}{c}} {{\mathrm{L}} - {\mathrm{S}}} \\ {{\mathrm{L}} - {\mathrm{I}}} \\ {{\mathrm{L}} - {\mathrm{R}}} \\ {{\mathrm{H}} - {\mathrm{S}}} \\ {{\mathrm{H}} - {\mathrm{I}}} \\ {{\mathrm{H}} - {\mathrm{R}}} \end{array}\left[ {\begin{array}{*{20}{c}} {\mathrm{1}} & {\mathrm{0}} & {\mathrm{0}} & {\mathrm{0}} & {\mathrm{0}} & {\mathrm{0}} \\ {\mathrm{0}} & {\mathrm{1}} & {\mathrm{0}} & {\mathrm{0}} & {\mathrm{0}} & {\mathrm{0}} \\ {\mathrm{0}} & {\mathrm{0}} & {\mathrm{1}} & {\mathrm{0}} & {\mathrm{0}} & {\mathrm{0}} \\ {\mathrm{0}} & {\mathrm{0}} & {\mathrm{0}} & {\mathrm{1}} & {\mathrm{0}} & {\mathrm{0}} \\ {\mathrm{0}} & {\mathrm{0}} & {\mathrm{0}} & {\mathrm{0}} & {\mathrm{1}} & {\mathrm{0}} \\ {\mathrm{0}} & {\mathrm{0}} & {\mathrm{0}} & {\mathrm{0}} & {\mathrm{0}} & {\mathrm{1}} \end{array}} \right]$$

In Eq. (21), *sex.ratio* was the average sex ratio and *litter.size* the average litter size in our study population. Each entry in *fecundity* was the elementary fecundity from a starting combination of social and infection states, to an ending combination of social and infection states.

We built four submatrices to account for the survival of cubs, subadults, breeders and non-breeders, respectively: **Survival**_C_**, Survival**_SA_**, Survival**_B_ and **Survival**_NB_ (Eqs. (22)–(25)). The survival matrices were diagonal, representing the survival probabilities (parameter *ϕ*) of cubs, subadults, breeders and non-breeders in given social and infection states, independently of transitions made during the projection interval. These matrices were as follows:

22 $${{\mathbf{Survival}}_{\mathrm{C}}} = \begin{array}{*{20}{c}} {{\mathrm{L}} - {\mathrm{S}}} \\ {{\mathrm{L}} - {\mathrm{I}}} \\ {{\mathrm{L}} - {\mathrm{R}}} \\ {{\mathrm{H}} - {\mathrm{S}}} \\ {{\mathrm{H}} - {\mathrm{I}}} \\ {{\mathrm{H}} - {\mathrm{R}}} \end{array}\left[ {\begin{array}{*{20}{c}} {\varphi {\mathrm{CLS}}} & {\mathrm{0}} & {\mathrm{0}} & {\mathrm{0}} & {\mathrm{0}} & {\mathrm{0}} \\ {\mathrm{0}} & {\varphi {\mathrm{CLI}}} & {\mathrm{0}} & {\mathrm{0}} & {\mathrm{0}} & {\mathrm{0}} \\ {\mathrm{0}} & {\mathrm{0}} & {\mathrm{0}} & {\mathrm{0}} & {\mathrm{0}} & {\mathrm{0}} \\ {\mathrm{0}} & {\mathrm{0}} & {\mathrm{0}} & {\varphi {\mathrm{CHS}}} & {\mathrm{0}} & {\mathrm{0}} \\ {\mathrm{0}} & {\mathrm{0}} & {\mathrm{0}} & {\mathrm{0}} & {\varphi {\mathrm{CHI}}} & {\mathrm{0}} \\ {\mathrm{0}} & {\mathrm{0}} & {\mathrm{0}} & {\mathrm{0}} & {\mathrm{0}} & {\mathrm{0}} \end{array}} \right]$$

23 $${{\mathbf{Survival}}_{\mathrm{SA}}} = \begin{array}{*{20}{c}} {{\mathrm{L}} - {\mathrm{S}}} \\ {{\mathrm{L}} - {\mathrm{I}}} \\ {{\mathrm{L}} - {\mathrm{R}}} \\ {{\mathrm{H}} - {\mathrm{S}}} \\ {{\mathrm{H}} - {\mathrm{I}}} \\ {{\mathrm{H}} - {\mathrm{R}}} \end{array}\left[ {\begin{array}{*{20}{c}} {\varphi {\mathrm{SALS}}} & {\mathrm{0}} & {\mathrm{0}} & {\mathrm{0}} & {\mathrm{0}} & {\mathrm{0}} \\ {\mathrm{0}} & {\varphi {\mathrm{SAIR}}} & {\mathrm{0}} & {\mathrm{0}} & {\mathrm{0}} & {\mathrm{0}} \\ {\mathrm{0}} & {\mathrm{0}} & {\varphi {\mathrm{SAIR}}} & {\mathrm{0}} & {\mathrm{0}} & {\mathrm{0}} \\ {\mathrm{0}} & {\mathrm{0}} & {\mathrm{0}} & {\varphi {\mathrm{SAHS}}} & {\mathrm{0}} & {\mathrm{0}} \\ {\mathrm{0}} & {\mathrm{0}} & {\mathrm{0}} & {\mathrm{0}} & {\varphi {\mathrm{SAIR}}} & {\mathrm{0}} \\ {\mathrm{0}} & {\mathrm{0}} & {\mathrm{0}} & {\mathrm{0}} & {\mathrm{0}} & {\varphi {\mathrm{SAIR}}} \end{array}} \right]$$

24 $${{\mathbf{Survival}}_{\mathrm{B}}} = \begin{array}{*{20}{c}} {{\mathrm{L}} - {\mathrm{S}}} \\ {{\mathrm{L}} - {\mathrm{I}}} \\ {{\mathrm{L}} - {\mathrm{R}}} \\ {{\mathrm{H}} - {\mathrm{S}}} \\ {{\mathrm{H}} - {\mathrm{I}}} \\ {{\mathrm{H}} - {\mathrm{R}}} \end{array}\left[ {\begin{array}{*{20}{c}} {\varphi {\mathrm{B}}} & {\mathrm{0}} & {\mathrm{0}} & {\mathrm{0}} & {\mathrm{0}} & {\mathrm{0}} \\ {\mathrm{0}} & {\varphi {\mathrm{B}}} & {\mathrm{0}} & {\mathrm{0}} & {\mathrm{0}} & {\mathrm{0}} \\ {\mathrm{0}} & {\mathrm{0}} & {\varphi {\mathrm{B}}} & {\mathrm{0}} & {\mathrm{0}} & {\mathrm{0}} \\ {\mathrm{0}} & {\mathrm{0}} & {\mathrm{0}} & {\varphi {\mathrm{B}}} & {\mathrm{0}} & {\mathrm{0}} \\ {\mathrm{0}} & {\mathrm{0}} & {\mathrm{0}} & {\mathrm{0}} & {\varphi {\mathrm{B}}} & {\mathrm{0}} \\ {\mathrm{0}} & {\mathrm{0}} & {\mathrm{0}} & {\mathrm{0}} & {\mathrm{0}} & {\varphi {\mathrm{B}}} \end{array}} \right]$$

25 $${{\mathbf{Survival}}_{\mathrm{NB}}} = \begin{array}{*{20}{c}} {{\mathrm{L}} - {\mathrm{S}}} \\ {{\mathrm{L}} - {\mathrm{I}}} \\ {{\mathrm{L}} - {\mathrm{R}}} \\ {{\mathrm{H}} - {\mathrm{S}}} \\ {{\mathrm{H}} - {\mathrm{I}}} \\ {{\mathrm{H}} - {\mathrm{R}}} \end{array}\left[ {\begin{array}{*{20}{c}} {\varphi {\mathrm{NB}}} & {\mathrm{0}} & {\mathrm{0}} & {\mathrm{0}} & {\mathrm{0}} & {\mathrm{0}} \\ {\mathrm{0}} & {\varphi {\mathrm{NB}}} & {\mathrm{0}} & {\mathrm{0}} & {\mathrm{0}} & {\mathrm{0}} \\ {\mathrm{0}} & {\mathrm{0}} & {\varphi {\mathrm{NB}}} & {\mathrm{0}} & {\mathrm{0}} & {\mathrm{0}} \\ {\mathrm{0}} & {\mathrm{0}} & {\mathrm{0}} & {\varphi {\mathrm{NB}}} & {\mathrm{0}} & {\mathrm{0}} \\ {\mathrm{0}} & {\mathrm{0}} & {\mathrm{0}} & {\mathrm{0}} & {\varphi {\mathrm{NB}}} & {\mathrm{0}} \\ {\mathrm{0}} & {\mathrm{0}} & {\mathrm{0}} & {\mathrm{0}} & {\mathrm{0}} & {\varphi {\mathrm{NB}}} \end{array}} \right]$$

As cubs were either susceptible or infected and never diagnosed as recovered in our original data set^10^, in the last step of the model development we deleted the two columns and two rows corresponding to recovered cubs. We thus ended up with a 22 × 22 meta-matrix **M** (Eq. (1)).

### Asymptotic analyses of the matrix population model

We used R v. 3.5.0. (R Core Team 2017)^41^. We used the function ‘is.matrix_irreducible’ in R’s package *popdemo* v 1.3-0^42^ to verify that the meta-matrix was irreducible. Matrix models are termed irreducible when their associated life cycles contain the transition rates to facilitate pathways from all states to all other states. Irreducible matrices are ergodic: the stable asymptotic growth rate is independent from the initial stage structure in the population projection. Both conditions should ideally be met for further analyses^43^.

When **M**(*t*) = **M** is considered constant (i.e., no time dependence, no stochasticity), the behaviour of such a deterministic model is well-known^22^. The modelled population is then characterised by an asymptotic growth rate and a stable asymptotic distribution over the 22 states, which are the dominant eigenvalue and the corresponding right eigenvector, respectively^22^. We used the functions ‘stable.state’ and ‘reproductive.value’ in R’s package *popbio* 2.4.4^44^ to determine the stable stage distribution and the reproductive values, based on the definitions provided by^22^.

We used the functions ‘pop.projection’ and ‘stage.vector.plot’ from R’s package *popbio* 2.4.4^44^ to plot the short-term population dynamics and the convergence to the stable stage distribution (Fig. 4), using the initial state probabilities estimated from the MECMR model (see Supplementary Results) as initial stage vector. The first function calculates the population growth rate and stable stage distribution by repeated projections of the Eq. (2).

We included a modification in the approach developed by^14^ to estimate the basic reproduction number *R*_0_ of this CDV during the epidemic period, considering only hyena-to-hyena transmission (i.e. no interspecific interactions as discussed). Our modified version of *R*_0_ accounted for the population’s growth rate and by doing this, we replaced the assumption of a stationary host population by accounting for observed population dynamics and thus also for changes in the proportion of infected individuals. As for equivalent time-continuous matrix models^31^, *R*_0_ is the dominant eigenvalue of the next generation matrix, itself being the product of the fundamental matrix by the reproductive matrix. The entries in the reproductive matrix in an epidemiological context correspond to the rate at which new infections are produced by infected individuals. In our case, there is only one infected state, thus only one type of infection is produced by only one type of (infected) individuals. We detail our modified approach below.

The starting point is the decomposition of the change in the number of infected in transmission and transitions:

26 $${\mathbf{A}} = {\mathbf{T}} + {\mathbf{R}}$$

where **A** is the projection matrix, **T** is the transition matrix and **R** is the reproductive matrix. Under asymptotic conditions, the changes in one time step are within a population that grows at rate *λ*. To represent the change in the number of infected as a change in proportion relative to the overall population, one can discount for population growth at rate *λ* and write the contribution matrix **C** to the “next generation of infected” (i.e. our modification of *R*_0_) as:

27 $${\mathbf{C}} = \lambda ^{ - 1}{\mathbf{R}} + \lambda ^{ - 1}{\mathbf{R}}\lambda ^{ - 1}{\mathbf{T}} + \lambda ^{ - 1}{\mathbf{R}}\lambda ^{ - 2}{\mathbf{T}}^2 + \lambda ^{ - 1}{\mathbf{R}}\lambda ^{ - 3}{\mathbf{T}}^{3 + } \ldots$$

which reduces to:

28 $${\mathbf{C}} = \lambda ^{ - 1}{\mathbf{R}}({\mathbf{I}} - \lambda ^{ - 1}{\mathbf{T}})^{ - 1} = R(\lambda {\mathbf{I}}-{\mathbf{T}})^{ - 1}$$

### Sensitivity analyses of *λ*, *R*_0_ and stochasticity

To determine which parameters contributed most to *λ* and *R*_0_ and predict the results of future changes in parameter estimates, we performed sensitivity analyses (Figs. 2 and 3). When elements of a population matrix are composed of several vital rates, the classical first order sensitivity analysis is not recommended, as it does not allow one to disentangle the effects of demographic, social and infection parameters. We therefore conducted lower-level sensitivity analyses for *λ* and *R*_0_. For *λ* we applied the function ‘vitalsens’ from the R package *popbio* 2.4.4^44^. For *R*_0_, we used a numerical approach in which we varied each parameter by 0.1% while maintaining the others constant, and calculated *R*_0_ at each iteration as previously described.

To account for parameter uncertainty (Figs. 1, 2, 3 and 5), we used Monte Carlo iterations. At each successive period (pre-epidem, epidem, post-epidem, plus the second post-epidemic period (2000–2010)), we simulated 1000 block-matrices **M**, implemented with the biological parameter estimates from the MECMR model (Table 1). First, we drew 1000 values from normal distributions with means equal to the regression coefficients of the MECMR model and with standard deviations equal to the standard errors associated with these regression coefficients. Sampling correlations among estimates were sufficiently small to be neglected. To obtain the biological parameter estimates and insure that they corresponded to probabilities bounded between 0 and 1, we back-transformed those simulated regression coefficients using the logit-function after accounting for the structural interactions and the temporal additive effects detected on those parameters. We could then describe temporal changes in abundance (Fig. 5) given parameter uncertainty during the 20 years of survey. For Fig. 5, we predicted population sizes for the next 10 years (2010–2020) by considering the 1000 block-matrices **M** implemented with the parameter estimates associated with the second post-epidemic period (2000–2010) and by determining the population vector of the number of individuals in the 22 demographic, social and infection states during the last year of the survey (2010). This vector was defined as the product of the mean abundance estimated in 2010 and the stable stage distribution. We then multiplied the matrices with this population vector to obtain 1000 population vectors and calculate the confidence intervals of the abundance the following year. These population vectors were then multiplied again by the simulated matrices to calculate the mean abundance and its associated confidence interval in the following year. This Markov chain in which the population vectors of the next year only depend on the population vectors of the current year and of the simulated projection matrices was then reiterated for 10 years. The starting population size was set to 100 individuals, equivalent to the size of the study population.

### Extension of the MECMR model to include temporal effects

Here, we adapted the MECMR model developed in Marescot et al.^10^ to include temporal effects on the survival and infection probabilities. The MECMR model was built in the software E-SURGE 1-9-0^45^. We tested whether including an additive effect of four periods (1990–1992: pre-epidem; 1993–1994: epidem, 1995–1999: post-epidem) and a further post-epidemic period (2000–2010) improved the model’s ranking, and used the parameter estimates for the first three periods (presented Table 1) as input for the stage-structured matrix population model. We assumed that the other parameters (i.e., transition probabilities among demographic states and among social states) did not vary across periods. Including temporal effects on survival and infection probabilities improved the model’s ranking substantially (Supplementary Results). As the best-ranked model from Marescot et al.^10^ produced some non-estimable parameters, we used the second best-ranked.

To obtain the model without effects of social status (Fig. 5, blue curve), we used the second best-ranked model and removed the formulation of the effect of social status on the relevant processes of (1) initial states, (2) survival, (3) infection, (4) breeding (demography), (5) social transition and (6) detection probabilities.

### Parameter estimates

All parameter estimates from the MECMR model used as in input for the matrix population model are provided Table 1 for the first three epidemic periods (pre-epidem, epidem, post-epidem). For survival and transition probabilities for the period 2000–2010, initial state, detection and assignment probabilities, see Supplementary Results.

The sex-ratio at birth is balanced and was estimated in a previous study to be equal to 0.52^46^. Here we used this estimate as input value for the parameter *sex.ratio* (Table 1, fecundity matrix, Eq. (21)). This parameter estimate had no standard error associated with it. We set an arbitrary standard error value equal to 0.03 for the sensitivity analyses.

In a previous study, we estimated average litter size based on observations conducted in our three main study clans between 1987 and 2006^47^ and the monitoring of the fate of 1124 spotted hyena cubs from 735 litters, comprising 351 (47.8%) singleton, 379 (51.6%) twin and five (0.7%) triplet litters. Thus, for the average litter size (parameter *litter.size*, Table 1, fecundity matrix, Eq. (21)) we used the value 1.53 (1124/735). This parameter estimate had no standard error associated with it. We set an arbitrary standard error value equal to 0.03 for the sensitivity analyses.

### Code availability

The R programming code to replicate all our analyses and figures is available from Github: https://github.com/LucileMarescot/SIR-Matrix-model. It is divided in three main sections: (1) code *1_Model construction* loads the input values for the three epidemic periods and builds the matrix model, (2) code *2_Model analysis_asymptotic* presents the asymptotic analysis of the matrix model and (3) code 3*_Model analysis_stochastic* presents the stochastic analysis of the matrix model. Each section is fully annotated so that each step can be replicated.

## Electronic supplementary material

Supplementary Information

## Data Availability

The original data from Marescot et al. (2018) supplied the parameter estimates used in this study and are archived in figshare^48^: 10.6084/m9.figshare.5840970. The parameter estimates used in this study are provided in Table 1 and in Supplementary Results.
