# Supplementary material for: Slow recovery from a disease epidemic in the spotted hyena, a keystone social carnivore
Source: Commun Biol. 2018 Nov 20;1:201. doi: 10.1038/s42003-018-0197-1 (PMC6244218; doi:10.1038/s42003-018-0197-1)
Supplement: Supplementary file 1 — Supplementary Information [file 42003_2018_197_MOESM1_ESM.pdf]

1. Supplementary Figures

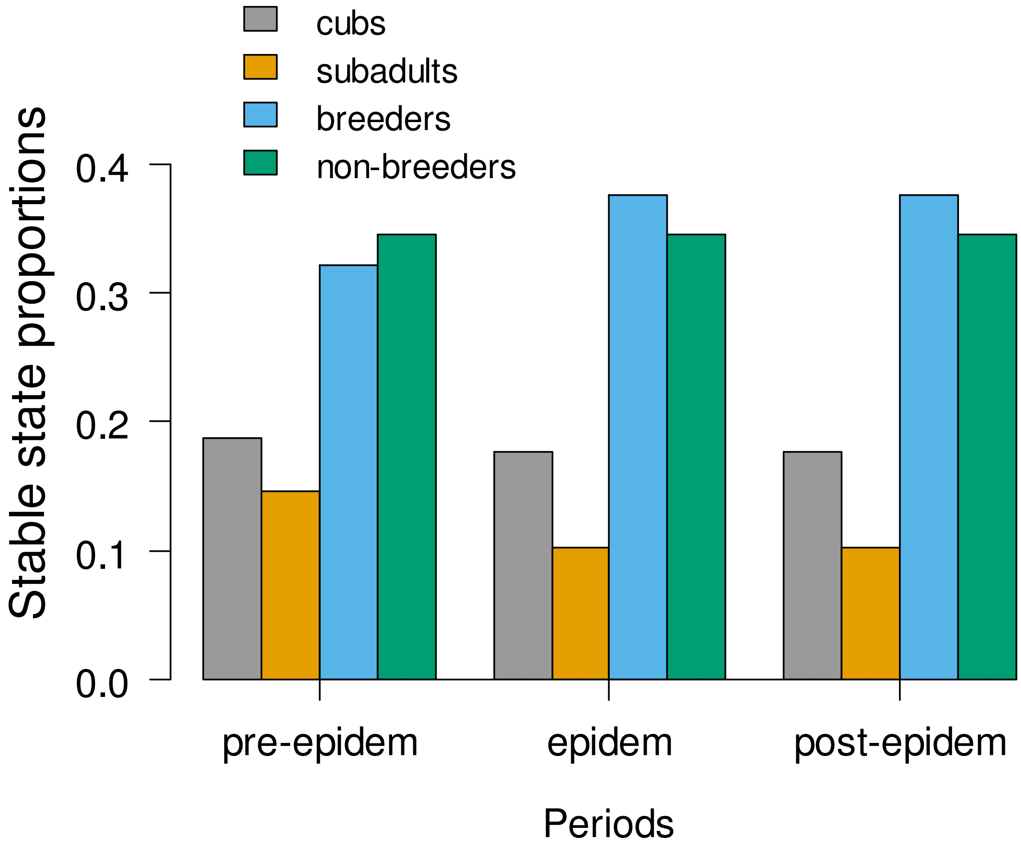

Supplementary Figure 1. Stable state proportions of female hyenas as cubs, subadults, breeders and non-breeders during each period.

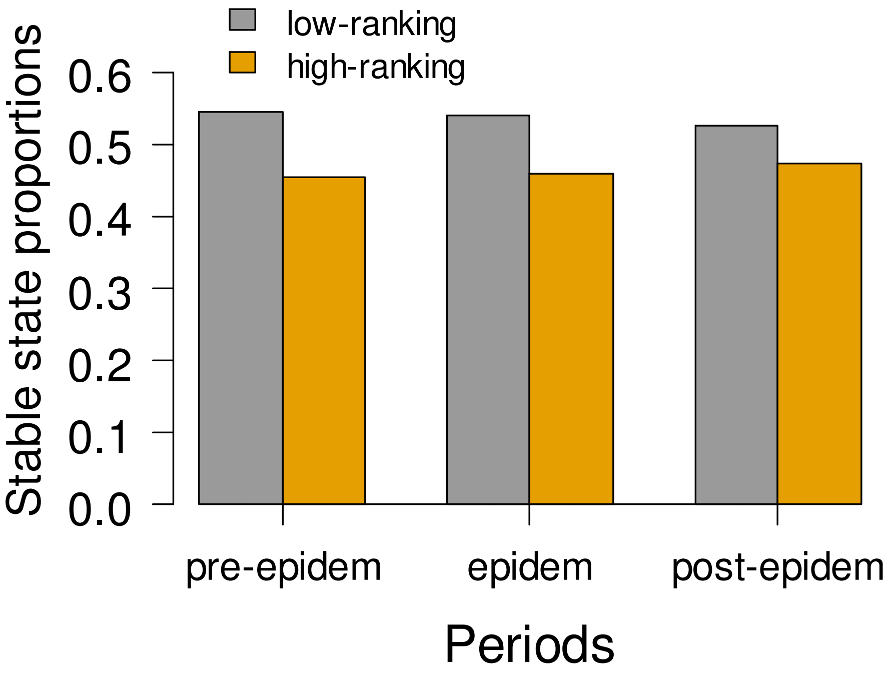

Supplementary Figure 2. Stable state proportions of high-ranking and low-ranking female hyenas during each period.

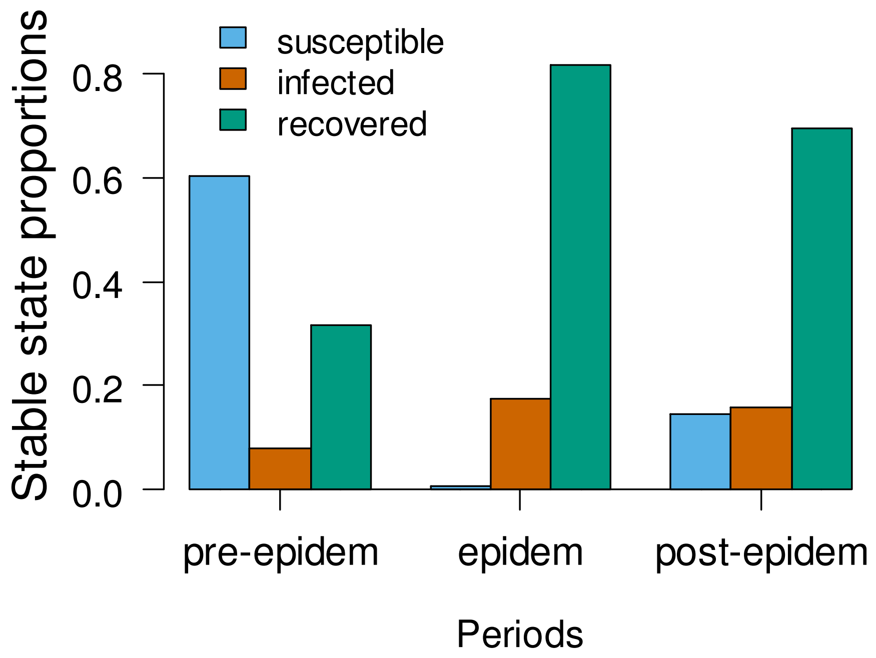

Supplementary Figure 3. Stable state proportions of female hyenas in terms of susceptible, infected and recovered during each period.

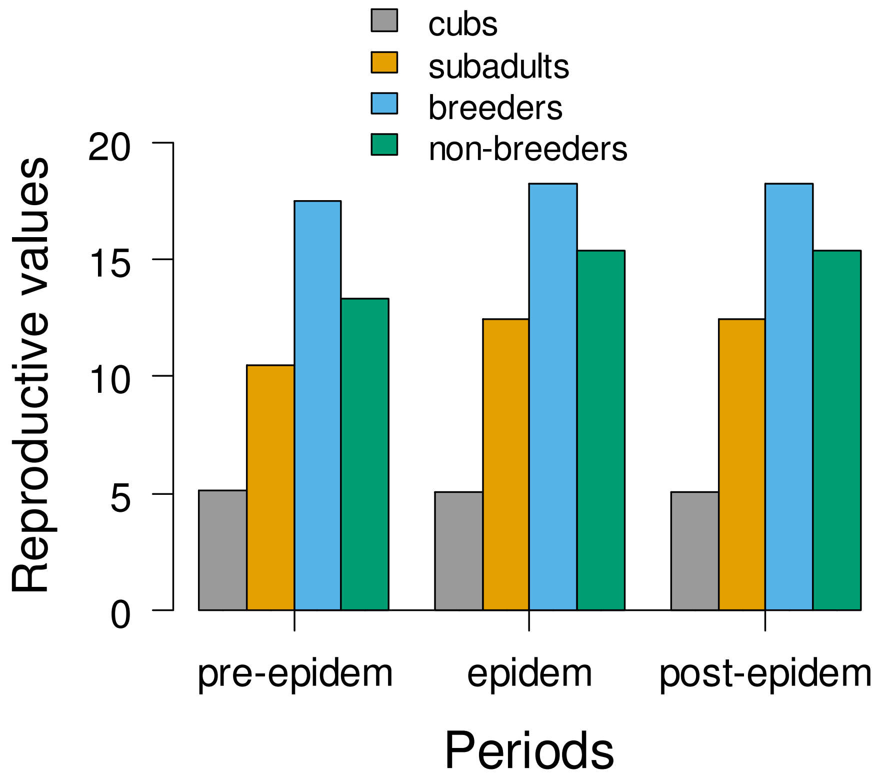

Supplementary Figure 4. Reproductive values of females from different demographic states during each period.

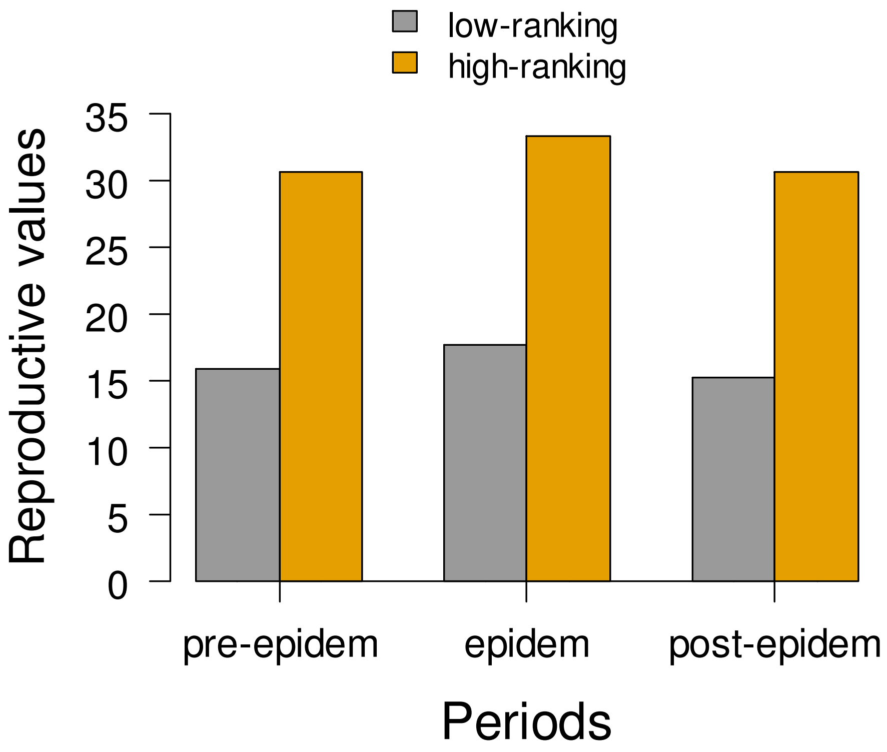

Supplementary Figure 5. Reproductive values of females of different social states during each period.

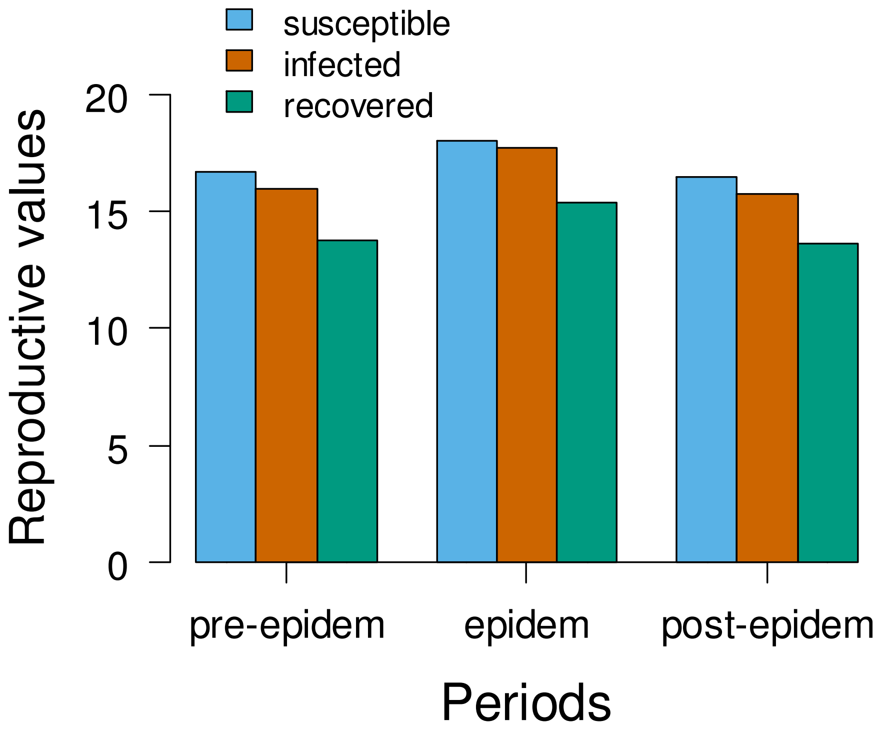

Supplementary Figure 6. Reproductive values of females of different infection states during each period.

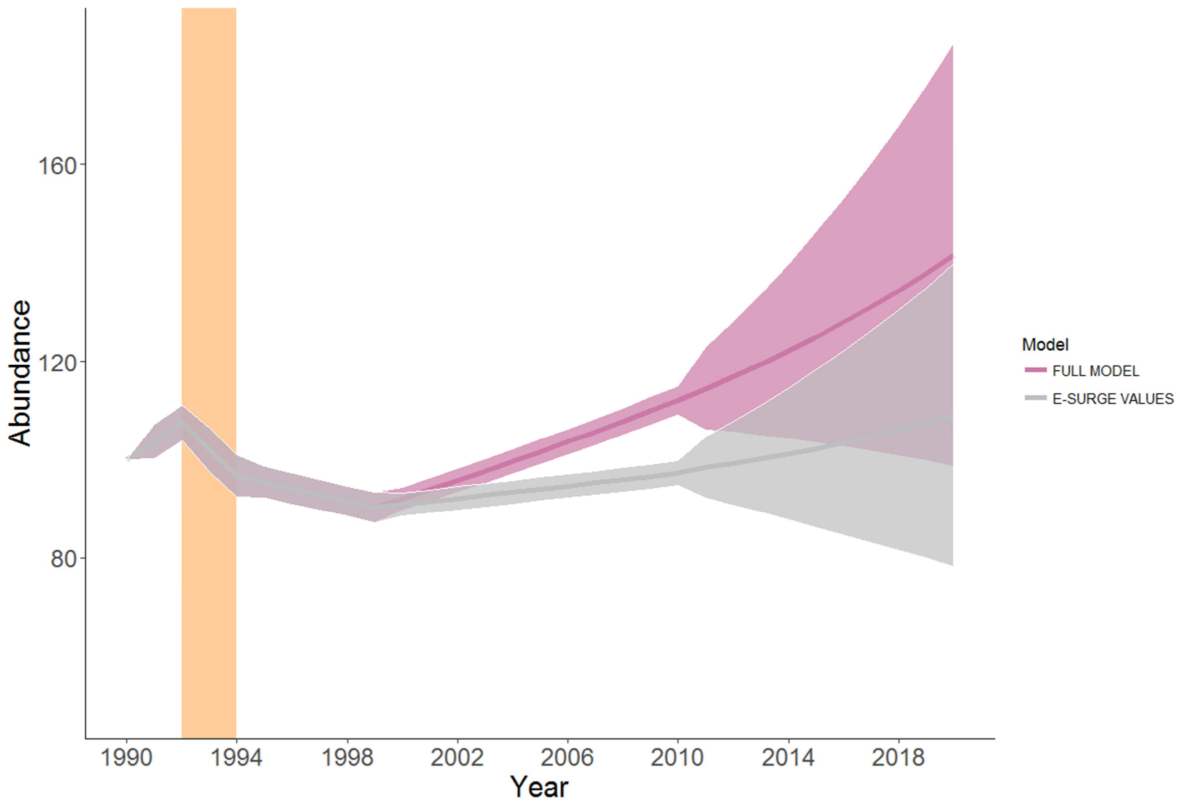

Supplementary Figure 7. Mean abundance (± 95 % confidence intervals) of female spotted hyenas projected throughout the study period (1990-2010) and predicted beyond it (2010-2020) based on the full model (pink) and a model where we use the probabilities of infection β estimated by E-SURGE during the period 2000-2010 (grey). The vertical bar (light orange) represents the period of 1993-1994 when the CDV epidemic occurred. This figure does not illustrate the actual number of hyenas in the study population; the projected population growth rate estimates from each period were used to produce this figure. The starting abundance was indexed as 100.

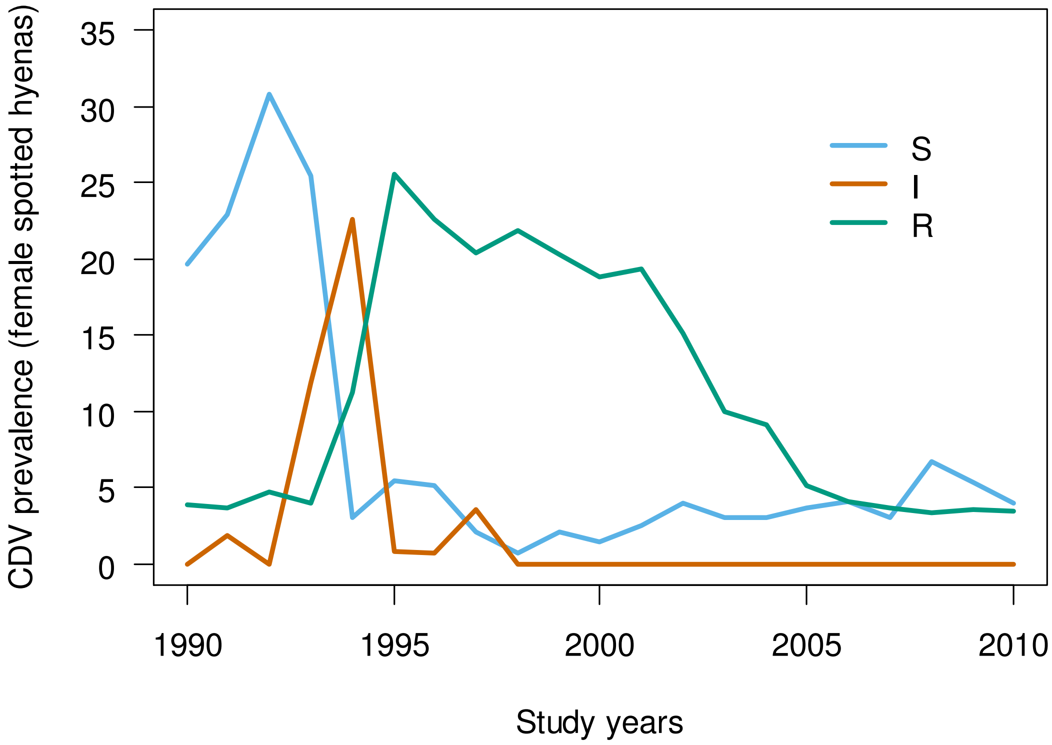

Supplementary Figure 8. CDV prevalence: Changes in the percentage of susceptible [S] (blue), infected [I] (orange) and recovered [R] (green) female clan members throughout the entire study period (1990-2010). Note that the percentage females in an unknown state are not shown.

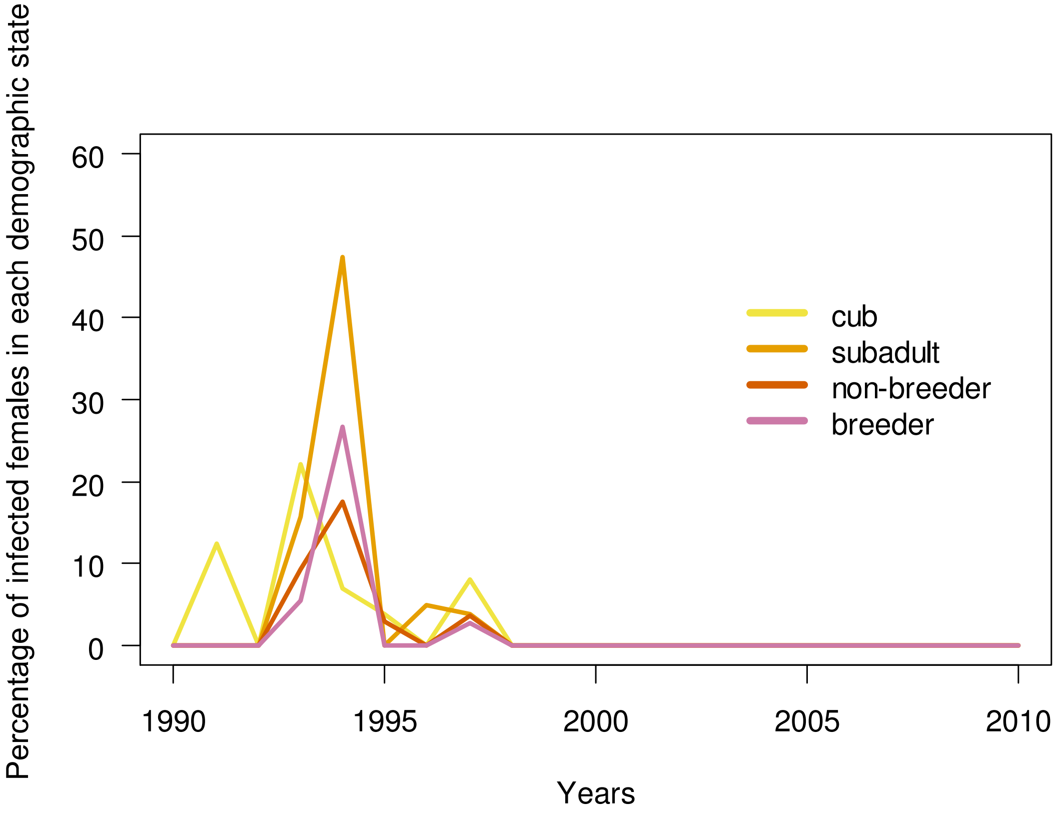

Supplementary Figure 9. Changes in the perecentage of infected [I] female clan members throughout the entire study period (1990-2010), in the different demographic states considered in the model; cubs (yellow), subadults (light orange), non-breeder (dark orange) and breeder (pink) per number of cubs, subadults, non-breeders and breeders each year, respectively. Note that females with unknown infection states are not shown.

2. Supplementary Tables

Supplementary Table 1. How infection states were assigned. Sample sizes in terms of number of individuals (*N* individuals) and number of (infection) states (*N* states) are provided for females. Reproduced from Marescot et al. [1]

	Serology (CDV antibodies)	Clinical sign	Virology (PCR screening)	♀		
					N individuals	*N* states
**Susceptible**	Seronegative	No sign	Vironegative	5	38	195
	Seronegative	No sign	Unknown	8		
	Unknown	No sign	Vironegative (cubs)	25		
**Infected**	Seropositive or Unknown	Sign observed	Unknown	52	54	54
	Seropositive or Unknown	Sign observed	Viropositive	0		
	Seropositive or Unknown	No sign	Viropositive	2		
**Recovered**	Seropositive	No sign	Unknown	4	12	314
	Seropositive	No sign	Vironegative	8		

Supplementary Table 2: State definition, abbreviation and sample sizes for each combination of a given demographic, social and infection state, as used in the female CMR data set. The infection state was assigned with uncertainty and thus included an unknown infection state. The grey shaded rows indicate that the sample size for the given combination of demographic, social and infection states was < 8. These combinations of states were not tested separately during the model selection procedure; instead they were pooled with other social or infection states to increase the global sample size. Reproduced from Marescot et al. [1]

Demographic states	Social states	Infection states	Abbreviated state combination	*N* ♀
Cub (C): 0−365 days	Low (L)	Susceptible (S)	C.L.S	31
		Infected (I)	C.L.I	5
		Recovered (R)	C.L.R	0
		Unknown (U)	C.L.U	201
	High (H)	Susceptible (S)	C.H.S	45
		Infected (I)	C.H.I	8
		Recovered (R)	C.H.R	0
		Unknown (U)	C.H.U	249
Subadult (SA): 366−720 days	Low (L)	Susceptible (S)	SA.L.S	5
		Infected (I)	SA.L.I	6
		Recovered (R)	SA.L.R	3
		Unknown (U)	SA.L.U	160
	High (H)	Susceptible (S)	SA.H.S	19
		Infected (I)	SA.H.I	8
		Recovered (R)	SA.H.R	1
		Unknown (U)	SA.H.U	223
Non-Breeder (NB): have not given birth to a litter	Low (L)	Susceptible (S)	NB.L.S	23
		Infected (I)	NB.L.I	3
		Recovered (R)	NB.L.R	53
		Unknown (U)	NB.L.U	409
	High (H)	Susceptible (S)	NB.H.S	26
		Infected (I)	NB.H.I	9
		Recovered (R)	NB.H.R	79
		Unknown (U)	NB.H.U	383
Breeder (B): have given birth to a litter	Low (L)	Susceptible (S)	B.L.S	21
		Infected (I)	B.L.I	7
		Recovered (R)	B.L.R	77
		Unknown (U)	B.L.U	302
	High (H)	Susceptible (S)	B.H.S	25
		Infected (I)	B.H.I	8
		Recovered (R)	B.H.R	101
		Unknown (U)	B.H.U	358

3. Supplementary Methods

This section is a detailed description of all the methods that were applied by Marescot et al.[1] to assess multi-event capture-mark-recapture (MECMR) parameter estimates. This section is structured as follows: (1) Fieldwork methods, (2) Labwork methods, (3) Data preparation methods and (4) Modelling.

1) <u>Fieldwork methods</u>

Step 1a − Identifying, aging, sexing and recording behavioural interactions between individuals in the field

From 1990 to 2010, three hyena clans located at the centre of the Serengeti NP were continuously monitored[2, 3]. All female clan members were identified by their unique spot patterns, scars and other characteristics such as ear notches. All females within a radius of about 100 m of communal dens were recorded for approximately three hours at dawn and dusk[4]. Cubs may be born at the communal den or at private birth dens, in which case they are usually brought to the communal den when about 7-14 days of age[5].

Clans contained philopatric females, which breed throughout the year[6], and their offspring, plus breeding males that are mostly immigrants[7]. Females and their offspring were socially dominant over immigrant males[4]. Females and natal males were first detected and aged within their first few weeks of life as previously detailed (e.g. [4,8]). Sex was assessed at approximately 3 months of age using the dimorphic glans morphology of the erect phallus following Frank et al.[9]. Weaning occurs at 12-20 months of age[6,10].

To avoid heterogeneity in the sampling frame, observations of individuals encountered in locations other than at or close to communal dens and birth dens were excluded. Members of study clans were habituated to the presence of observers in vehicles, which enabled detailed observations of social interactions and clinical signs indicative of CDV, even in very young individuals.

To construct strictly linear dominance hierarchies in each clan we recorded submissive behaviours during dyadic adult female-female interactions (e.g. [4, 11]). Interactions were recorded ad libitum during frequent observation periods of approximately three hour duration at both dawn and dusk, mostly at clan dens and during all-night observations.

Step 1b − Monitoring clinical signs of CDV

For health monitoring and disease diagnosis, we recorded the start and end of clinical signs consistent with (but not exclusive to) clinical signs of CDV infection (blue sheen on eyes, respiratory distress, ataxia, poor limb coordination, bloody faeces and listlessness) and of secondary infections know to typically occur in spotted hyenas infected with CDV, such as nasal and or ocular discharge[12]. Some

spotted hyenas suspected to have contracted CDV showed several signs. Individuals with such signs were particularly closely monitored to record their potential date of death or disappearance.

Step 1c − Collecting biological samples (faeces, blood, tissues)

We screened for the shedding of CDV by RT-PCR (for details see below) in faecal (n = 61) and saliva samples (n = 2) collected non-invasively, and blood (n = 27), or tissue (n = 16) samples collected from known hyenas that died from natural causes, were hit by vehicles on roads or were immobilized mostly for the removal of wire snares set by bushmeat hunters[13].

2) <u>Labwork methods</u>

Step 2a − Serological analyses

CDV antibody titers in sera were measured using assays established at four laboratories between 1994 and 2015: (1) Animal Health Diagnostic Centre, College of Veterinary Medicine, Cornell University, Ithaca NY 14853, USA; (2) Department of Infectious Diseases, Institute of Virology, Veterinary University Hannover, Germany; (3) Leibniz Institute for Zoo and Wildlife Research, Berlin, Germany; (4) Diagnostic Laboratory, Institute of Virology, University of Giessen, Giessen, Germany). The titre levels considered to indicate significant exposure to CDV were set by each laboratory, and were as follows: for (1) Serum-neutralizing antibody test, titres > 1:10 were considered positive, for (2) and (3) Neutralizing-Peroxydase-linked antibody assay, titres > 40 were considered positive and (4) Serum neutralization test, titres > 0.5 log ND50 were considered positive.

Step 2b − RT-PCR screening

Following collection, faecal samples were thoroughly mixed and divided into aliquots. Faecal, tissue and blood samples prior to 2004 were frozen in liquid nitrogen and then stored and transported at -80 °C. Samples collected after 2004 were preserved in RNAlater (Sigma–Aldrich Co, St. Louis, MO, USA) following the manufacturer's instructions, stored and transported at -10 °C. All samples were later stored at -80 °C until further use.

Unless stated otherwise, all kits were used according to manufacturer's instructions. Viral RNA was isolated from samples using the MinElute virus spin Kit (Qiagen, Hilden, Germany). RNA from whole blood or WBC was isolated using TRI Reagent® and the Direct-zol™ RNA MiniPrep (Zymo Research Europe, Freiburg, Germany) following manufacturer's instructions and including an in-column DNase I treatment. Reverse transcriptase-polymerase chain reaction (RT-PCR) was performed using SuperScript™ III One-Step RT-PCR System (Life Technologies GmbH, Darmstadt, Germany) using primers 5 (5'- TGCTGGAGATGGTTTAATTCAATC-3') and 12 (5'-TAGAGGAGACCAGGTCACTGT-3') targeting a 514-nt fragment of the 3'-end of the haemagglutinin (H) gene. Amplicons were purified using Qiagen PCR purification Kit (Qiagen,

Hilden, Germany). Sequencing was bidirectional and conducted using the fluorescent Big Dye Terminator Cycle sequencing Kit 3.1 (ABI, Darmstadt, Germany). Sequences were analyzed on an ABI model 3130xl Genetic Analyzer (ABI). Editing of the sequences was carried out with BIOEDIT v.7.0.9.0[14]. A BLAST search at the NCBI server (http://blast.ncbi.nlm.nih.gov/Blast.cgi) was performed to find out whether the sequence fragments obtained matched known CDV sequences.

3) <u>Data preparation methods</u>

Step 3a − Creating encounter histories

To meet the assumptions of capture-mark-recapture (CMR) models, only systematic observations of female clan members at communal dens and birth dens were included, as indicated previously. For this purpose, multiple observations of females during a given year were synthesized into a single yearly summary: any female clan member that was observed at its clan communal and/or birth dens at least once throughout the year was set as "detected" that year. This data set includes information on the detection (presence) or non-detection (putative absence, possibly death) of any female for every year between 1990 and 2010.

Step 3b − Assigning demographic states

Females (n = 625) were classified as cubs (C), subadults (SA), breeders (B) or non-breeders (NB). Female cubs were younger than 1 year. Subadult females were aged from 1 to less than 2 years. Adults were 2 years and older. Female breeders gave birth to a litter during a given year, as documented by a freshly ruptured clitoris caused by parturitions[2] and/or subsequent lactation, whereas non-breeders did not. The vast majority of female breeders were lactating females; hence, this state represented the elevated energetic cost of lactation[15].

Step 3c − Assessing standardized social status and assigning social states

Females were classified as either high-ranking (H) or low-ranking (L), based on their positions in the (strictly linear) clan-specific adult female dominance hierarchy. Dominance hierarchies were adjusted after each loss or recruitment of adults and when dyadic interaction data revealed than an individual had increased or fallen in rank. To permit the comparison of the ranks held by individuals within hierarchies containing different numbers of animals within and across clans and years, we computed for each rank held by an individual during its life-time a standardized rank. This measure places the ranks within a given hierarchy evenly between the highest (standardized rank: +1) and the lowest (standardized rank: −1) rank[11,16]. For breeder and non-breeder females, the social states were high-ranking (H: average standardized rank ranging from 0.01 to +1) and low-ranking (L: average standardized rank ranging from -1 to 0). If different social states were observed for an individual within a year, we assigned the most frequently observed state (i.e. H or L) during that year for that individual.

Step 3d − Assigning infection states

Infection states were assigned using three diagnostic procedures: (i) RT-PCR screening for the presence or absence of CDV RNA in samples; results were classified as 'viropositive' or 'vironegative', respectively, (ii) CDV antibody titres in sera; results were classified 'seropositive' when serum contained a significant antibody titre against CDV and 'seronegative' when not, and (iii) the observation of clinical signs associated with CDV infection in hyenas, and the secondary infections it causes in this species, hereafter termed 'clinical signs'. Individuals were assigned as:

- Susceptible (S): individuals with a seronegative result, unless clinical signs were observed and/or a viropositive result obtained during the same year. Cubs with a vironegative result were also considered susceptible unless clinical signs and/or a viropositive result were observed during the same year. We assumed that we would not have missed any clinical sign in cubs since they are under particularly detailed observations at communal dens.
- Infected (I): individuals with clinical signs and/or a viropositive result. This state encompassed both the non-contagious and contagious stages of CDV infection.
- Recovered (R): individuals with a seropositive result without clinical signs and/or a viropositive result during the same year.
- Unknown (U): individuals lacking both RT-PCR screening or serological results, and in which clinical signs were not observed.

As for all morbilliviruses, individuals that survive CDV infection acquire lifelong immunity (e.g. [12, 17-24]). For this reason CDV infection occurs only once in an individual's life. By applying this fact, any individual classified as (i) 'susceptible' in a given year was classified as 'susceptible' during all previous years when the individual was detected; (ii) 'infected' in a given year was classified as 'susceptible' during all previous years, and 'recovered' during all subsequent years when the individual was detected; (iii) 'recovered' in a given year was classified as 'recovered' during all subsequent years when the individual was detected.

Sample sizes in terms of number of individuals and number of states for (1) each infection state and for (2) each combination of demographic, social and infection state are provided in Supplementary Table 1 and Supplementary Table 2, respectively.

4) <u>Modelling</u>

Step 4a − Building the model

We used a multi-event CMR (MECMR) model[25] fitted in E-SURGE 1.9.0.[26] to estimate survival probabilities and state transition probabilities (the "biological processes"). This model permits the estimation of such parameters whilst simultaneously accounting for potential methodological biases. These include gaps between monitoring periods, left-censored data when individuals were observed

first as adults at the beginning of the study, and right-censored data when individuals were still alive but not necessarily detected at the end of the study (the "observation processes"; see [27-29]). Individuals detected ('captured') in a given year were assigned an (S, I or R) infection state if data were available, or an unknown infection state (U) if data were unavailable[30]. More classical models would normally discard individuals with unknown infection states, but this would most likely result in biases (as shown in e.g. [31]). We assumed that known infection states were assigned correctly, i.e. we deliberately ignored potential errors in the assignment of infection states [30, 32]. The biological processes included survival (ϕ) and transition probabilities. The infection probability β was the probability for a susceptible individual to become infected, r was the probability of staying in the same social state and the breeding probability ψ was the probability that subadult, breeder and non-breeder females became breeder females. The biological processes were the product of 4 squared matrices representing transitions between demographic states, social states, infection states and survival.

Transitions between demographic states: The matrix *Demo* (1) considers the transitions of females to three demographic states; subadults (SA), breeders (B) or non-breeders (NB):

$$Demo = \begin{array}{c} C \\ SA \\ B \\ NB \end{array} \begin{bmatrix} 0 & 1 & 0 & 0 \\ 0 & 0 & \psi & 1-\psi \\ 0 & 0 & \psi & 1-\psi \\ 0 & 0 & \psi & 1-\psi \end{bmatrix} \qquad (1)$$

with Ψ the transition probability to the B state accessible from SA, B and NB females and with $1- \Psi$ its complement. Each entry in *Demo* is the probability of transition from a 'starting' demographic state (4 rows corresponding to the demographic states C, SA, B, NB on the left side of the matrix) to the 'following' demographic state (4 columns corresponding to the demographic states C, SA, B, NB, not shown for simplicity). Here for example, surviving cubs (C) ('starting state') have a transition probability to the subadult state (SA) ('following state') that is equal to 1. Please note that Ψ is a symbolic notation here. This parameter could vary between states depending on the model being tested.

Transitions between social states: The matrix *Social* (2) considers the transitions of females to two social states, High social state (H) or Low social state (L), as females can either remain or change their social state:

$$Social = \begin{matrix} L \\ H \end{matrix} \begin{bmatrix} r & 1-r \\ 1-r & r \end{bmatrix}$$

(2)

with *r* the probability of staying in the same social state and (1- *r*) its complement. Each entry in *Social* is the probability of transition from a 'starting' social state (2 rows corresponding to the social states L and H) to a 'following social state (2 columns corresponding to the social states L and H, not shown for simplicity). Please note that *r* is a symbolic notation here. This parameter could vary between states depending on the model being tested.

Transitions between infection states: The matrix *Infection* (3) considers the transitions of females to three infection states, susceptible (S), infected (I) and recovered (R):

$$Infection = \begin{matrix} S \\ I \\ R \end{matrix} \begin{bmatrix} 1-\beta & \beta & 0 \\ 0 & 0 & 1 \\ 0 & 0 & 1 \end{bmatrix}$$

(3)

with β the infection probability (i.e. the probability of transition from a susceptible to an infected state) and *1-* β its complement. Each entry in *Infection* is the probability of transition from a 'starting' infection state category (3 rows corresponding to the infection states S, I, R) to a 'following' infection state (3 columns corresponding the infection states S, I, R, not shown for simplicity). Please note that β is a symbolic notation here. This parameter could vary between states depending on the model being tested.

Survival: The matrix *Survival* (4) accounts for the annual survival probabilities of females, shows annual apparent survival probabilities and is:

$$Survival = \begin{matrix} C \\ SA \\ B \\ NB \\ Dd \end{matrix} \begin{bmatrix} \phi & 0 & 0 & 0 & 1-\phi \\ 0 & \phi & 0 & 0 & 1-\phi \\ 0 & 0 & \phi & 0 & 1-\phi \\ 0 & 0 & 0 & \phi & 1-\phi \\ 0 & 0 & 0 & 0 & 1 \end{bmatrix}$$

(4)

with ϕ the survival probability. Each entry in *Survival* is the probability of surviving from a 'starting' demographic state (4 rows corresponding to the demographic states C, SA, B, NB on the left side of the matrix). *Dd* represents the transition to the "dead" state. Please note that ϕ is a symbolic notation here. This parameter could vary between states depending on the model being tested.

To represent all possible transitions between demographic, social, and infection states of surviving individuals we then combined these matrices in the following way.

Modeling the initial state probabilities: For females the matrix $\boldsymbol{I_S}$ (5) is the matrix of the initial state probabilities, composed of a single row with 24 states, representing the different combinations of demographic, social and infection states (the 'dead' state cannot appear as an initial state). This vector provides information about the proportion of females in different combinations of demographic, social and infection states in the female CMR data set.

$$\boldsymbol{Is} = [\pi\ \pi\ \pi\ \pi\ \pi\ \pi\ \pi\ \pi\ \pi\ \pi\ \pi\ \pi\ \pi\ \pi\ \pi\ \pi\ \pi\ \pi\ \pi\ \pi\ \pi\ \pi\ \pi\ \pi\ (1-\pi)] \quad (5)$$

with π being the probability of being in a given initial demographic, social and infection state. The entries in I_s correspond to the probabilities (from left to right) of being in a combination of states in the sequence presented in Supplementary Table 2, i.e. from C.L.S to B.H.R (without the U states).

Combining all biological processes:

The matrix ***Infection /Social*** (6) then combines the infection matrix with the social matrix, and displays the possible transitions to the different infection states within and between the two social states:

$$\textbf{\textit{Infection/Social}} = \begin{array}{c} L.S \\ H.S \\ L.I \\ H.I \\ L.R \\ H.R \end{array} \begin{bmatrix} (1-\beta)*r & (1-\beta)*(1-r) & \beta*r & \beta*(1-r) & 0 & 0 \\ (1-\beta)*(1-r) & (1-\beta)*r & \beta*(1-r) & \beta*r & 0 & 0 \\ 0 & 0 & 0 & 0 & r & (1-r) \\ 0 & 0 & 0 & 0 & (1-r) & r \\ 0 & 0 & 0 & 0 & r & (1-r) \\ 0 & 0 & 0 & 0 & (1-r) & r \end{bmatrix} \quad (6)$$

Each entry in ***Infection/Social*** is the probability of transition from a 'starting' combination of a social (L and H) and an infection (S, I and R) state, with six rows corresponding to the combinations L.S, H.S, L.I, H.I, L.R, H.R on the left side of the matrix, to an 'ending' combination of social and infection states, with six columns corresponding to the combinations L.S, H.S, L.I, H.I, L.R, H.R (not shown for simplicity). The * symbol represents the multiplication of parameter estimates.

The final matrix $\boldsymbol{Final_{females}}$ represent the transition probabilities of surviving females to successive demographic states given their specific social and infection states as formulated in ***Infection/Social*** (6). Each element representing the transition probability of C, SA, B and NB (for females) represents a sub-matrix of dimension ***Infection/Social***. $\boldsymbol{Final_{females}}$ (7) is thus a matrix of total dimensions 25 by 25 as it considers demographic, social and infection states and the absorbing death state. The × symbol represents a multiplication of matrices. We also implemented a diagonal matrix of 1, denoted

as *Diag*(1), with similar dimension as ***Infection/Social*** to better represent the extension of both the survival and demography matrices when combining all matrices into the two final matrix of $\boldsymbol{Final_{females}}$ (7).

$$\textbf{Final}_{females} = \begin{matrix} C \\ SA \\ B \\ NB \\ Dd \end{matrix} \begin{bmatrix} \phi \times Infection/Social & 0 & 0 & 0 & 1-\phi \\ 0 & \phi \times Infection/Social & 0 & 0 & 1-\phi \\ 0 & 0 & \phi \times Infection/Social & 0 & 1-\phi \\ 0 & 0 & 0 & \phi \times Infection/Social & 1-\phi \\ 0 & 0 & 0 & 0 & 1 \end{bmatrix} \times \begin{bmatrix} 0 & Diag(1) & 0 & 0 & 0 \\ 0 & 0 & \psi \times Diag(1) & (1-\psi) \times Diag(1) & 0 \\ 0 & 0 & \psi \times Diag(1) & (1-\psi) \times Diag(1) & 0 \\ 0 & 0 & 0 & (1-\psi) \times Diag(1) & 0 \\ 0 & 0 & 0 & 0 & 1 \end{bmatrix}$$

(7)

$$\textbf{Diag(1)} = \begin{bmatrix} 1 & 0 & 0 & 0 & 0 & 0 \\ 0 & 1 & 0 & 0 & 0 & 0 \\ 0 & 0 & 1 & 0 & 0 & 0 \\ 0 & 0 & 0 & 1 & 0 & 0 \\ 0 & 0 & 0 & 0 & 1 & 0 \\ 0 & 0 & 0 & 0 & 0 & 1 \end{bmatrix}$$

(8)

Modeling the observation process:

Detection: The detection matrices accounted for the probability of detection p and the probability of non-detection 1-p of females in different demographic, social and infection states. Each element of $\boldsymbol{Det_{Df}}$ is multiplied by a diagonal matrix of 1, $\boldsymbol{Diag}(\mathbf{1})$ of dimension ***Infection/Social*** to later extend the $\boldsymbol{Det_{Df}}$ matrix across the social and infection states. Multiplying each transition by the diagonal matrix $\boldsymbol{Diag}(\mathbf{1})$, with similar dimension as ***Infection/Social***, reflects the extension of the detection matrix across the social and infection ones:

$$\boldsymbol{Det_{Df}} = \begin{matrix} C \\ SA \\ B \\ NB \\ Dd \end{matrix}\begin{bmatrix} (1-p)*Diag(1) & p*Diag(1) & 0 & 0 & 0 \\ (1-p)*Diag(1) & 0 & p*Diag(1) & 0 & 0 \\ (1-p)*Diag(1) & 0 & 0 & p*Diag(1) & 0 \\ (1-p)*Diag(1) & 0 & 0 & 0 & p*Diag(1) \\ 1 & 0 & 0 & 0 & 0 \end{bmatrix} \qquad (9)$$

Assignment of infection states: The partial observation matrix ***PO*** (10) below represents cases where individuals are detected but their infection state is unknown. This is characterized by the last column, whose elements 1-δ denote the probability of failing to assign an infection state to an individual that has been detected. Each element of the matrix is multiplied by a diagonal matrix of 1, $\boldsymbol{Diag}(\mathbf{1})$ of dimension 8 by 8 for females. Such an extension of the ***PO*** matrix represents the partial observation of infection states across the demographic and social states:

$$\boldsymbol{PO} = \begin{matrix} S \\ I \\ R \end{matrix}\begin{bmatrix} \delta*Diag_2(1) & 0 & 0 & (1-\delta)*Diag_2(1) \\ 0 & \delta*Diag_2(1) & 0 & (1-\delta)*Diag_2(1) \\ 0 & 0 & \delta*Diag_2(1) & (1-\delta)*Diag_2(1) \end{bmatrix} \qquad (10)$$

The multiplication of the detection matrix by the partial observation matrix then provided the final matrix for the observation process (not shown), reflecting the potential incomplete detection of individuals as well as the potential uncertainty on individual infection states.

Example of encounter history formulation: Finally, we illustrate an observed encounter history, formulated for a fictive female detected for three consecutive years following birth. The fictive female was detected as a high-ranking cub at the first sampling occasion and assigned to be 'susceptible' (denoted as C.H.S), then detected as a high-ranking subadult with an unknown disease state the following year (SA.H.U) and finally detected as low-ranking non-breeder with an unknown disease state, (NB.L.U). The true encounter history of this individual could have been one of three possibilities:

(1) C.H.S → SA.H.S → NB.L.S

(2) C.H.S → SA.H.S → NB.L.I

(3) C.H.S → SA.H.I → NB.L.R

The probability of observing the illustrated encounter history is equivalent to the following formulation:

$$
\begin{aligned}
&\mathrm{P(C.H.S, SA.H.U, NB.L.U)} = \\
&\phi_1^C r_1^H (1-\beta_1) p_1^C \delta_1^S \phi_2^{SA} (1-r_2^H)(1-\beta_2)(1-\psi_2^{SA\to B}) p_2^{SA} (1-\delta_2^S) p_3^{NB} (1-\delta_3^S) + \\
&\phi_1^C r_1^H (1-\beta_1) p_1^C \delta_1^S \phi_2^{SA} (1-r_2^H)(\beta_2)(1-\psi_2^{SA\to B}) p_2^{SA} (1-\delta_2^S) p_3^{NB} (1-\delta_3^I) + \\
&\phi_1^C r_1^H (\beta_1) p_1^C \delta_1^S \phi_2^{SA} (1-r_2^H)(1-\psi_2^{SA\to B}) p_2^{SA} (1-\delta_2^I) p_3^{NB} (1-\delta_3^R)
\end{aligned}
\tag{11}
$$

where the indexed number *1* represents the first sampling occasion (i.e. the first detection occasion), *2* the second and *3* the third, ,ϕ the annual survival probability, *r*, the probability of staying in the same social state, (1- *r* being its complement), β, the infection probability (i.e. the probability of transition: susceptible → infected, with 1- β being its complement), ψ the transition probability to the B state (with the upper index SA→B denoting the specific transition from subadult to breeder, i.e., breeding probability of primiparous females); with 1- ψ being the complement), *p* the detection probability (1-*p* being its complement, i.e. the probability that an individual is not detected) and δ the assignment probability of an individual into an infection state (susceptible, infected or recovered) conditional on its detection (1- δ being its complement, i.e., the probability that an individual has its infection state not assigned, i.e. that it is 'unknown').

Step 4b − Goodness of fit

We performed a goodness-of-fit (GOF) test to (1) determine whether our data met the assumptions of CMR models and (2) validate our model[33]. This test allows us to check the validity of the model, by verifying that the "detected section of the population" is representative of the section of the population that is undetected. More specifically, this test allows to verify that the fate and the detection of a given individual is (1) independent from the fate and the detection of any other individual, and that it is not influenced by (2) the monitoring design or the (3) behaviour of that individual [34, 35]. Any violation in these assumptions can lead to the underestimation of the variance of parameters and result in the selection of potentially biased (over-parameterized) models[34, 35].

To verify that we fulfilled these assumptions, we conducted a preliminary assessment of the GOF of the most parameterized model to the data, by calculating chi-square values in contingency tables using the software U-CARE 2.3.2[35].

The validation of our model was first done with the test '3G.SM'. This overall composite test is based on the conjunction of several null hypotheses depicting the main assumptions stated above. We did not find any significant departure from those assumptions, meaning that the fully parameterized model adequately fitted the data ($\chi^2 = 75.69$, df = 143 p-value = 0.7).

We then performed more specific tests. The test '3G.SR' is aimed at detecting a potential lack-of-fit due to heterogeneity in individual detection or the presence of individuals that temporarily move beyond the core study area. If the presence of such transient individuals on the study site is overlooked, survival will be underestimated[35]. The null hypothesis states that "there is no difference in the probability of being later re-detected between "new" and "old" individuals detected simultaneously"[35]. The test result was not significant ($\chi^2 = 13.7$, df = 32, p-value = 0.99), showing that our estimates are not biased by potential heterogeneity in individual detection or temporal absence from the study area.

The test 'M.ITEC' for 'trap-dependence' is aimed at detecting a potential lack-of-fit due to the monitoring design resulting in some individuals responding positively or negatively to the capturing/detection method (e.g.. when some individuals are attracted to baited camera traps or afraid of flashing cameras). It tests the null hypothesis that "there is no difference in the probabilities of being re-encountered in the different states at the occasion $_{i+1}$ between the animals in the same state at occasion $_{\mathrm{i}}$ whether encountered or not encountered at this date, conditional on presence at both occasions"[35]. We could not run this test because the great majority of females were re-detected the year following their first detection, resulting in insufficient variation in the re-detection probability to run this test. However, our close observations of thousands of hyenas at communal dens during the past thirty years do not suggest in any way that some clan members would have avoided the vicinity of communal dens owing to the presence of the research vehicle. We are confident that our monitoring design did not influence the probability of re-detecting a female after its first detection.

Finally, the test 'WBWA' (WhereBeforeWhereAfter) is aimed at verifying that the movements of animals are not based on the knowledge of previously visited sites[34]. This test is typically used in multi-site CMR models that record encounters with marked animals (very often bird species that establish breeding colonies) over different sites. In multi-state CMR models, 'sites' correspond to 'states'. Such a 'memory effect' would constitute a violation of the assumptions of CMR models because it can lead to different behaviours (and hence biological states) for individuals belonging to the same set of animals detected on a given occasion, depending on which states they had previously assumed[34]. This test is based on the null hypothesis that 'there is no difference in the expected state of next re-encounter among individuals previously encountered in the different states"[35, 36]. In our case, a non-significant test would confirm that

the state transition of an individual detected at a given occasion does not depend on its state at the previous occasion. The test result was not significant (χ^2 =165.5, df = 172, p-value = 0.62), showing that there was no effect of past state.

Step 4c − Model selection

We fitted a set of candidate models for females. It is often recommended to parameterize the observation process (here: detection and assignment) before the biological one (e.g. [37]). Starting with a constant-only model, we sequentially parameterized the: (1) assignment of infection states, (2) detection, (3) survival, (4) infection, (5) social transitions and (6) breeding transitions, testing for effects of social, demographic and infection states on those processes.

For the observation processes, we chose models with the lowest value of the quasi-Akaike information criterion corrected for small sample size (QAICc [38]). QAICc was used rather than the common AIC in order to correct for potentially autocorrelated and overdispersed data[38]. For the biological processes, best models were those with the largest number of parameters within a range of 2 QAICc units of difference from the model with the lowest QAICc value, in order to avoid dismissing potentially important biological predictors of interest. We considered that fully identifiable models differing by less than 2 QAICc units from the model with the lowest QAICc might have substantial empirical support for explaining variation in the response variable[39].

4. Supplementary Results

In this section, we present the QAICc values of the MECMR models that include temporal effects (i.e. full model and model without social structure in Fig. 5) and the estimates of the probabilities of initial states, detection and assignment for the full model and the model without social structure. We also show the results obtained for the second post-epidemic period between 2000 and 2010 (i.e. parameter estimates and population growth rate) for these different types of models.

1) <u>Model ranking</u>

The QAICc of the MECMR model presented in the main text was 9978.7. This model was better ranked than the equivalent model without any temporal effects as presented in Marescot et al.[1] (QAICc = 10476.4, 1st row in Table 1 of Marescot et al.[1]). The temporal model without social structure had a QAICc of 9999.8.

2) <u>Parameter estimates</u>

a) Time-invariant

The initial state, detection and assignment probabilities did not vary across periods. The initial state probabilities depended on an interaction between demographic and social states (maximum likelihood estimate and associated standard error (s.e.m.): $\pi^{C.H} = 0.48$, $\pi^{C.L} = 0.38$, $\pi^{SA.H} = 0.02$, $\pi^{SA.L} = 0.01$, $\pi^{B.H} = 0.02$, $\pi^{B.L} = 0.02$, $\pi^{NB.H} = 0.03$, $\pi^{NB.L} = 0.04$). This indicates that at first detection, there was a predominance of cubs as compared with other demographic states and a pre-dominance of high-ranking females over low-ranking females. In the model without social structure, the initial state probabilities depended on demographic states ($\pi^{C} = 0.86$, $\pi^{SA} = 0.02$, $\pi^{B} = 0.04$, $\pi^{NB} = 0.07$).

The detection probabilities varied with female social status, irrespective of a female's demographic or infection state. High-ranking females were slightly more often detected than low-ranking ones (high-ranking: $p^{H} = 1.00$ (s.e.m. < 0.01), low-ranking: $p^{L} = 0.98 \pm 0.01$). In the model without social structure, the detection probability was constant ($p = 0.99$, s.e.m. < 0.01).

The assignment probabilities of S, I and R infection states were best predicted by a model including the effect of infection states (susceptible δ^{S}: 0.86 ± 0.07; infected δ^{I}: 0.16 ± 0.02; recovered δ^{R}: 0.14 ± 0.01). The same applies to the model without social structure.

b) Period 2000-2010

During the second post-epidemic period (2000-2010), the survival probabilities in the full model were as follows: high-ranking susceptible cubs 0.88 ± 0.05, low-ranking susceptible cubs 0.84 ± 0.07, high-ranking infected cubs 0.75 ± 0.05, low-ranking infected cubs 0.58 ± 0.07, high-ranking susceptible

subadults 0.96 ± 0.04, low-ranking susceptible subadults 0.88 ± 0.14, pooled high and low-ranking infected and recovered subadults 0.65 ± 0.03, high and low-ranking non-breeders 0.84 ± 0.01, high and low-ranking breeders 0.94 ± 0.01.

The MECMR estimated high values for the probability of infection β (high-ranking cubs: 0.77 ± 0.09, low-ranking cubs: 0.90 ± 0.06, high-ranking subadults, breeders and non-breeders: 0.53 ± 0.13, low-ranking subadults, breeders and non-breeders: 0.19 ± 0.08) for the period 2000-2010. We expected particularly low values of β during this period because our previous molecular analysis of the CDV strains in the Serengeti ecosystem indicated that the highly virulent strain responsible for the epidemic in 1993/1994 was not detected in any of our study clans after 1997 and in any carnivore after 1999[40]. In addition, the striking clinical signs of CDV infection that we observed in 1993/1994 and the associated high mortality in hyena juveniles[12] were never observed after 1999 in any of our study clans and there was no 'infected' state in our data set after 1997 (see [1]).

We suggest that these high estimated values are a likely consequence of an increase in uncertainty during this period, since the proportion of unknown infection states is far higher than during the 3 previous periods (pre-epidem, epidem, post-epidem) combined (total number of unknown states during the first 3 periods: n = 895 vs.1390 during the second post-epidemic period). Similarly, the number of known susceptible and recovered states declined (total number of susceptible states during the first 3 periods: n = 135 vs. 60 during the second post-epidemic period and recovered: n = 186 vs. 128).

To project changes in population size in Fig. 5 (full model, pink curve), we thus chose to explicitly set the probabilities of infection β to 0 for all demographic and social states during this second post-epidemic period, instead of using these high parameter values estimated by the software E-SURGE. We also set the probabilities of infection β to 0 for all demographic and social states during this second post-epidemic period for the model without social structure (blue curve in Fig 5). We show Supplementary Fig. 7 the projected changes in population size when using the probabilities of infection estimated by E-SURGE between 2000 and 2010 (grey curve).

For the full model presented Figure 5 in the main text, in pink, for which we set the probability of infection β equal to zero during the period 2000-2010, the population growth rate was $\lambda = 1.02 \pm 0.03$ during this period. The values of λ during the pre-epidemic, epidemic and first post-epidemic period (1995-1999) are illustrated Figure 1 in the main text. For the full model presented Supplementary Figure

7, in grey, for which we used the probabilities of infection β provided by the software E-Surge during the period 2000-2010, the population growth rate was $\lambda = 1.00 \pm 0.03$.

5. Supplementary References

1. Marescot, L. et al. Social status mediates the fitness costs of infection with canine distemper virus in Serengeti spotted hyenas. *Funct. Ecol.* **32**, 1237-1250 (2018).

2. Hofer, H. & East, M. L. The commuting system of Serengeti spotted hyaenas - how a predator copes with migratory prey. III. Attendance and maternal care. *Anim. Behav.* **46**, 575-589 (1993).

3. East, M. L., Otto, E., Helms, J., Thierer, D., Cable, J. & Hofer, H. Does lactation lead to resource allocation trade-offs in the spotted hyaena? *Behav. Ecol. Sociobiol.* **69**, 805-814 (2015)

4. Hofer, H. & East, M. L. Behavioral processes and costs of co-existence in female spotted hyenas: a life history perspective. *Evol. Ecol.* **17**, 315-331 (2003).

5. East, M., Hofer, H. & Türk, A. Functions of birth dens in spotted hyaenas (*Crocuta crocuta*). *J. Zool.* **219**, 690-697 (1989).

6. Hofer, H. & East, M. L. Population dynamics, population size, and the commuting system of Serengeti spotted hyenas in *Serengeti II: Dynamics, Management, And Conservation Of An Ecosystem*, (ed. Sinclair, A. R. E. & Arcese, P) 332-363 (University of Chicago Press, 1995).

7. East, M. L. & Hofer, H. Male spotted hyenas (*Crocuta crocuta*) queue for status in social groups dominated by females. *Behav. Ecol.* **12**, 558-568 (2001).

8. Hofer, H. & East, M. L. The commuting system of Serengeti spotted hyaenas - how a predator copes with migratory prey .I. Social organisation. *Anim. Behav.* **46**, 547-557 (1993).

9. Frank, L. G., Glickman, S. E. & Licht, P. Fatal sibling aggression, precocial development, and androgens in neonatal spotted hyenas. *Science* **252**, 702-704 (1991).

10. Holekamp, K. E., Smale, L. & Szykman, M. Rank and reproduction in the female spotted hyaena. *J. Reprod. Infertil.* **108**, 229-237 (1996).

11. East, M.L., Burke, T., Wilhelm, K., Greig, C. & Hofer, H. Sexual conflicts in spotted hyenas: male and female mating tactics and their reproductive outcome with respect to age, social status and tenure. *Proc. Biol. Sci.* **270**, 1247-1254 (2003).

12. Haas, L. *et al.* Canine distemper virus infection in Serengeti spotted hyaenas. *Vet. Microbiol.* **49**, 147-152 (1996).

13. Hofer, H., East, M. L., & Campbell, K. L. Snares, commuting hyaenas and migratory herbivores: humans as predators in the Serengeti. In *Symposia Of the Zoological Society of London*, pp. 347-366 (1993).

14. Hall, T.A. (1999) BioEdit: a user-friendly biological sequence alignment editor and analysis program for Windows 95/98/NT. *Nucleic Acids Symposium Series*, pp. 95-98.

15. Hofer, H., Benhaiem, S., Golla, W. & East, M. L. Trade-offs in lactation and milk intake by competing siblings in a fluctuating environment. *Behav. Ecol.* **27**, 1567-1578 (2016).

16. Goymann, W. et al. Social, state-dependent and environmental modulation of faecal corticosteroid levels in free-ranging female spotted hyenas. *Proc. Biol. Sci.* **268**, 2453-2459 (2001).

17. Appel, M.J. & Summers, B.A. Pathogenicity of morbilliviruses for terrestrial carnivores. *Vet. Microbiol.* **44**, 187-191 (1995).

18. Beineke, A., Puff, C., Seehusen, F. & Baumgärtner, W. Pathogenesis and immunopathology of systemic and nervous canine distemper. *Vet. Immunol.Immunopathol.***127**, 1-18 (2009).

19. Beineke, A., Baumgärtner, W. & Wohlsein, P. Cross-species transmission of canine distemper virus - an update. *One Health* **1**, 49-59 (2015).

20. Deem, S.L., Spelman, L.H., Yates, R.A. & Montali, R.J. Canine distemper in terrestrial carnivores: a review. *J. Zoo. Wild. Med.* **31**, 441-451 (2000).

21. Garenne, M., Leroy, O., Beau, J.-P. & Sene, I. Child mortality after high-titre measles vaccines: prospective study in Senegal. *The Lancet* **338**, 903-907 (1991).

22. Harrison, T.M. et al. Antibodies to canine and feline viruses in spotted hyenas (*Crocuta crocuta*) in the Masai Mara National Reserve. *J. Wild. Dis.* **40**, 1-10 (2004).

23. Sawatsky, B., Wong, X. X., Hinkelmann, S., Cattaneo, R. & von Messling, V. Canine distemper virus epithelial cell infection is required for clinical disease but not for immunosuppression. *J. Virol.* **86**, 3658-3666 (2012).

24. Tatsuo, H., Ono, N. & Yanagi, Y. Morbilliviruses use signaling lymphocyte activation molecules (CD150) as cellular receptors. *J.Virol.* **75**, 5842-5850 (2001).

25. Pradel, R. Multievent: an extension of multistate capture–recapture models to uncertain states. *Biometrics* **61**, 442-447 (2005).

26. Choquet, R., & Nogue, E. E-SURGE 1.8 user's manual. CEFE, UMR 5175, Montpellier, France (2011).

27. Schaub, M., Gimenez, O., Schmidt, B. R. & Pradel, R. Estimating survival and temporary emigration in the multistate capture-recapture framework. *Ecology* **85**, 2107-2113 (2004).

28. Lebreton, J. D., Nichols, J. D., Barker, R.J., Pradel, R. & Spendelow, J. A. (2009) Modeling individual animal histories with multistate capture–recapture models. *Adv.Ecol.Res*. **41**, 87-173.

29. Gimenez, O., Lebreton, J.-D., Gaillard, J.-M., Choquet, R. & Pradel, R. Estimating demographic parameters using hidden process dynamic models. *Theor. Popul. Biol.* **82**, 307-316 (2012).

30. Conn, P.B. & Cooch, E.G. Multistate capture–recapture analysis under imperfect state observation: an application to disease models. *J. Appl.Ecol.* **46**, 486-492 (2009).

31. Desprez, M., McMahon, C. R., Hindell, M. A., Harcourt, R., & Gimenez, O. Known unknowns in an imperfect world: incorporating uncertainty in recruitment estimates using multi-event capture–recapture models. *Ecol.Evol.* **3**, 4658-4668 (2013).

32. Chambert, T. et al. Exposure of black-legged kittiwakes to Lyme disease spirochetes: dynamics of the immune status of adult hosts and effects on their survival. *J. Anim.Ecol.* **81**, 986-995 (2012).

33. Grosbois, V. et al.. Assessing the impact of climate variation on survival in vertebrate populations. *Biol. Rev.* **83**, 357-399 (2008).

34. Pradel, R., O. Gimenez, & Lebreton, J-D. Principles and interest of GOF tests for multistate capture–recapture models. *Anim. Biodiv.Conserv.* **28**, 189-204 (2005).

35. Choquet, R., Lebreton, J.D., Gimenez, O., Reboulet, A.M. & Pradel, R. U-CARE: Utilities for performing goodness of fit tests and manipulating CApture-REcapture data. *Ecography* **32**, 1071-1074 (2009).

36. Pradel, R., Wintrebert, C.M. & Gimenez, O. A proposal for a goodness-of-fit test to the Arnason-Schwarz multisite capture-recapture model. *Biometrics* **59**, 43-53 (2003).

37. Culina, A., Lachish, S., Pradel, R., Choquet, R. & Sheldon, B.C. A multievent approach to estimating pair fidelity and heterogeneity in state transitions. *Ecol. Evol.***3**, 4326-4338 (2013).

38. Hannan, E.J. & Quinn, B.G. The determination of the order of an autoregression. *J. R. Stat. Soc. Series B* **41**, 190-195 (1979).

39. Burnham, K. P. & Anderson, D. R. *Model Selection And Multimodel Inference: A Practical Information-Theoretic Approach* (Springer Science & Business Media, Berlin, Heidelberg, New York, 2003).

40. Nikolin, V. M. *et al.* Canine distemper virus in the Serengeti ecosystem: molecular adaptation to different carnivore species. *Mol. Ecol.* **26**, 2111-2130 (2017).
